# Identification and Characterization of *Anaplasma phagocytophilum* Proteins Involved in Infection of the Tick Vector, *Ixodes scapularis*

**DOI:** 10.1371/journal.pone.0137237

**Published:** 2015-09-04

**Authors:** Margarita Villar, Nieves Ayllón, Katherine M. Kocan, Elena Bonzón-Kulichenko, Pilar Alberdi, Edmour F. Blouin, Sabine Weisheit, Lourdes Mateos-Hernández, Alejandro Cabezas-Cruz, Lesley Bell-Sakyi, Marie Vancová, Tomáš Bílý, Damien F. Meyer, Jan Sterba, Marinela Contreras, Nataliia Rudenko, Libor Grubhoffer, Jesús Vázquez, José de la Fuente

**Affiliations:** 1 SaBio, Instituto de Investigación en Recursos Cinegéticos IREC-CSIC-UCLM-JCCM, Ronda de Toledo s/n, 13005, Ciudad Real, Spain; 2 Department of Veterinary Pathobiology, Center for Veterinary Health Sciences, Oklahoma State University, Stillwater, Oklahoma, 74078, United States of America; 3 Centro Nacional de Investigaciones Cardiovasculares, Melchor Fernández Almagro 3, 28029, Madrid, Spain; 4 The Roslin Institute and Royal (Dick) School of Veterinary Studies, University of Edinburgh, Easter Bush, Midlothian, EH25 9RG, United Kingdom; 5 The Pirbright Institute, Ash Road, Pirbright, Woking, GU24 0NF, United Kingdom; 6 Center for Infection and Immunity of Lille (CIIL), INSERM U1019 – CNRS UMR 8204, Université Lille Nord de France, Institut Pasteur de Lille, Lille, France; 7 Institute of Parasitology, Biology Centre of the Academy of Sciences of the Czech Republic, Branišovská 31, České Budějovice, CZ-37005, Czech Republic; 8 Faculty of Science, University of South Bohemia, Branišovská 31, České Budějovice, CZ-37005, Czech Republic; 9 CIRAD, UMR CMAEE, Site de Duclos, Prise d’eau, F-97170, Petit-Bourg, Guadeloupe, France; 10 INRA, UMR1309 CMAEE, F-34398, Montpellier, France; Washington State University, UNITED STATES

## Abstract

*Anaplasma phagocytophilum* is an emerging zoonotic pathogen transmitted by *Ixodes scapularis* that causes human granulocytic anaplasmosis. Here, a high throughput quantitative proteomics approach was used to characterize *A*. *phagocytophilum* proteome during rickettsial multiplication and identify proteins involved in infection of the tick vector, *I*. *scapularis*. The first step in this research was focused on tick cells infected with *A*. *phagocytophilum* and sampled at two time points containing 10–15% and 65–71% infected cells, respectively to identify key bacterial proteins over-represented in high percentage infected cells. The second step was focused on adult female tick guts and salivary glands infected with *A*. *phagocytophilum* to compare *in vitro* results with those occurring during bacterial infection *in vivo*. The results showed differences in the proteome of *A*. *phagocytophilum* in infected ticks with higher impact on protein synthesis and processing than on bacterial replication in tick salivary glands. These results correlated well with the developmental cycle of *A*. *phagocytophilum*, in which cells convert from an intracellular reticulated, replicative form to the nondividing infectious dense-core form. The analysis of *A*. *phagocytophilum* differentially represented proteins identified stress response (GroEL, HSP70) and surface (MSP4) proteins that were over-represented in high percentage infected tick cells and salivary glands when compared to low percentage infected cells and guts, respectively. The results demonstrated that MSP4, GroEL and HSP70 interact and bind to tick cells, thus playing a role in rickettsia-tick interactions. The most important finding of these studies is the increase in the level of certain bacterial stress response and surface proteins in *A*. *phagocytophilum*-infected tick cells and salivary glands with functional implication in tick-pathogen interactions. These results gave a new dimension to the role of these stress response and surface proteins during *A*. *phagocytophilum* infection in ticks. Characterization of *Anaplasma* proteome contributes information on host-pathogen interactions and provides targets for development of novel control strategies for pathogen infection and transmission.

## Introduction

*Anaplasma phagocytophilum* (Rickettsiales: Anaplasmataceae) is a tick-borne pathogen that is the etiologic agent of human, canine and equine granulocytic anaplasmosis and tick-borne fever of ruminants [1–3]. Despite this organism being an emerging zoonotic pathogen in many regions of the world, vaccines are not available for prevention of transmission and infection of humans and animals [4]. While *A*. *phagocytophilum* infection may resolve without therapy, the pathogen has been shown to be susceptible to tetracycline antibiotics [4].

*A*. *phagocytophilum* is an intracellular bacterium that infects tick tissues such as gut and salivary glands and vertebrate host neutrophils [5–11]. While transcriptomics and proteomics analyses have contributed to our understanding of the mechanisms by which *A*. *phagocytophilum* infection affects host and vector gene expression and protein content [8–17], less information is available on bacterial molecular mechanisms involved in pathogen infection and multiplication [18–21]. Proteomics characterization of *Anaplasma* spp. provides information on host-pathogen interactions and suggests possible targets for the control of pathogen infection and transmission [17–26].

The transcriptome and/or proteome of *A*. *phagocytophilum* have been characterized in tick salivary glands during transmission feeding [21] and in human HL-60 cells [18–20]. The proteome of the closely related pathogen *A*. *marginale* was characterized in IDE8 and ISE6 tick cells [23, 24, 26]. These experiments demonstrated the existence of host-specific *Anaplasma* proteins that may be involved in bacterial infection and multiplication. However, the *Anaplasma* proteome has not been characterized in low and high percentage infected tick cells to identify proteins functionally important during bacterial multiplication in the tick vector.

The proteome is dynamic with each developmental stage presenting an ensemble of proteins that give rise to substantial diversity and thus the need to characterize changes as infection proceeds from low to high percentage infected tick cells. Although *in vivo* data might be more relevant to understand bacterial infection [21], *in vitro* studies allow for monitoring *A*. *phagocytophilum* infection for a better comparison between low and high percentage infected cells. Nevertheless, the experimental approach using tick cell cultures should be complemented with *in vivo* studies to identify bacterial proteins playing a relevant role during infection and multiplication in the tick vector.

The aim of this research was to identify *A*. *phagocytophilum* proteins involved in infection of the tick vector, *I*. *scapularis*. Our hypothesis was that *A*. *phagocytophilum* proteins that increase as infection proceeds in cultured tick cells and ticks may be important for infection. To address this hypothesis, we characterized *A*. *phagocytophilum* proteome during rickettsial multiplication in *I*. *scapularis* cultured tick cells, guts and salivary glands and demonstrated that this bacterium uses certain stress response and surface proteins to favor pathogen infection and multiplication. Characterization of *Anaplasma* proteome contributes information on host-pathogen interactions and also provides targets for development of novel control strategies for pathogen infection and transmission.

## Results and Discussion

### Changes in *A*. *phagocytophilum* proteome correlate with bacterial infection cycle in adult female tick guts and salivary glands

The transcriptome and proteome of *A*. *phagocytophilum* were previously characterized in *I*. *scapularis* tick salivary glands during transmission feeding with similar protein functions identified by both approaches [21]. *In vivo* data is more relevant to understand bacterial infection. However, the proteome is dynamic with each developmental stage presenting an ensemble of proteins that give rise to substantial diversity. Additionally, the limited amount of material that can be obtained *in vivo* may affect the detection of proteins present at low levels but playing an important role during bacterial life cycle. Therefore, it is important to characterize changes as infection increases from low to high percentage infected cells *in vitro* and *in vivo* in both guts and salivary glands. These tick tissues were selected for this study because guts and salivary glands play a major role during pathogen acquisition, multiplication and transmission [17].

**Fig 1:**
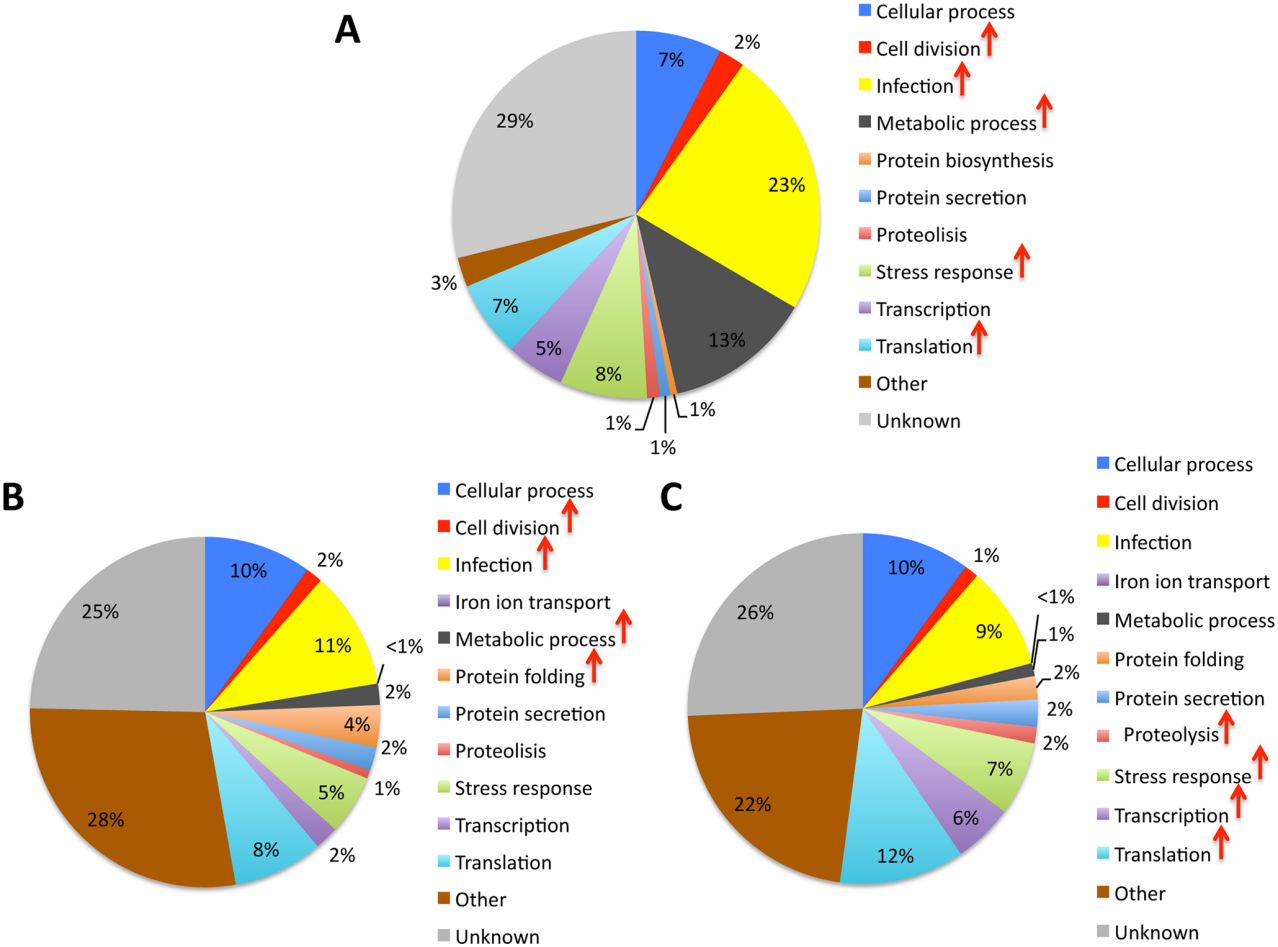
*A*. *phagocytophilum* protein ontology in infected tick cells and adult female guts and salivary glands. (A) Rickettsia cell protein ontology for biological process of differentially represented proteins in high percentage infected cells when compared to low percentage infected cells. (B) Rickettsia protein ontology for biological process of proteins identified in infected adult female guts. (C) Rickettsia protein ontology for biological process of proteins identified in infected adult female salivary glands. Only proteins identified with a FDR < 0.05 and at least 2 peptides per protein were included in the analysis. After discarding tick proteins, proteins with the same description in the Anaplasmataceae were grouped and the total number of PSM for each protein were normalized against the total number of PSM on each infected tick cell or tissue and compared between low and high percentage infected cells or between salivary glands and guts by Chi2-test (P = 0.05; N = 3 for tick cells and N = 2 for ticks). Biological processes with over-represented proteins in high percentage infected cells and in infected tick guts or salivary glands are indicated with red arrows (P<0.05).

The first step in this research was focused on *I*. *scapularis* embryonic ISE6 cells in which it is easy to select for low and high percentage *A*. *phagocytophilum*-infected cells, thus allowing for the identification of key bacterial proteins over-represented in high percentage infected cells when compared to cells infected at low percentage. A high throughput iTRAQ-based quantitative proteomics approach was used to characterize *A*. *phagocytophilum* differential protein representation in high percentage (65–71% infected cells) when compared to low percentage (10–15% infected cells) infected ISE6 tick cells. Infected tick cell cultures are asynchronous and therefore both late and early-infected cells are present in high percentage infected cells, but likely with more late than early-infected cells when compared to the low percentage infected cells. The statistical analysis of proteomics data revealed a total of 765 *Anaplasma* proteins at a false discovery rate (FDR) of 5% (S1 Table). Of them, 88 were differentially represented in high percentage infected cells when compared to low percentage infected cells (S1 Table). Analysis of protein ontology for bacterial differentially represented proteins showed that biological processes (BP) cell division, infection, metabolic process, stress response and translation were affected as infection proceeded in tick cells (Fig 1A).

The second step in this research was focused on adult female tick guts and salivary glands infected with *A*. *phagocytophilum* to compare *in vitro* results obtained under controlled conditions with those occurring during bacterial infection *in vivo*. The results of quantitative proteomics showed the identification of 1295 unique *Anaplasma* proteins in infected tick guts (N = 988) and/or salivary glands (N = 1248) (S2 Table). These proteins were identified with 2906 and 6696 peptide-spectrum matches (PSM) in tick guts and salivary glands, respectively (S2 Table). After grouping proteins with the same description, the analysis of protein ontology showed that while proteins in cell division, infection, metabolic process and protein folding BPs were over-represented in infected guts (Fig 1B), in infected salivary glands proteins in the proteolysis, stress response, transcription and translation BPs were over-represented (Fig 1C).

Of the proteins over-represented in high percentage infected ISE6 cells, major surface protein 4 (MSP4), 60 kDa chaperonin (GroEL) and chaperone protein DnaK (HSP70) were also over-represented in infected tick salivary glands when compared to tick guts (S1 and S2 Tables).

Previous characterization of the *A*. *phagocytophilum* proteome has shown that chaperones (GroEL), MSP2 (P44) surface, translation and stress response proteins are among the most abundant proteins found in *I*. *scapularis* tick salivary glands [21] while in human HL-60 cells, DNA replication proteins are more abundant [19, 20]. In the closely related species *A*. *marginale* grown in cultured tick cells, surface proteins including MSP4 are abundant in the bacterial proteome [23, 26], together with proteins involved in stress response (GroEL), transcription and translation [23]. These results supported previous reports that *A*. *phagocytophilum* transcription and translation are more active than replication in tick salivary glands during tick transmission feeding at both mRNA and protein levels [21]. Herein, the characterization of *A*. *phagocytophilum* proteome in infected tick guts and salivary glands agreed with these previous findings and showed that infection, cells division and metabolic proteins are more abundant in tick guts while proteins in the stress response, transcription and translation pathways are more abundant in tick salivary glands (Fig 1B and 1C). The more abundant proteins in infected cultured tick cells represented a combination of those observed in infected tick guts and salivary glands (Fig 1A). These results reflected the fact that infected tick cell cultures are asynchronous and correlated well with the developmental cycle of *A*. *phagocytophilum*, in which cells convert from an intracellular reticulated, replicative form to the nondividing infectious dense-core form [7, 20, 21].

### The *A*. *phagocytophilum* proteins over-represented in high percentage infected tick cells and salivary glands are surface-exposed

The *A*. *phagocytophilum* proteins GroEL, MSP4 and HSP70 over-represented in high percentage infected tick cells and infected salivary glands were selected for further characterization. These proteins are major surface proteins (MSP4) or members of the stress response pathway (GroEL and HSP70).

**Fig 2:**
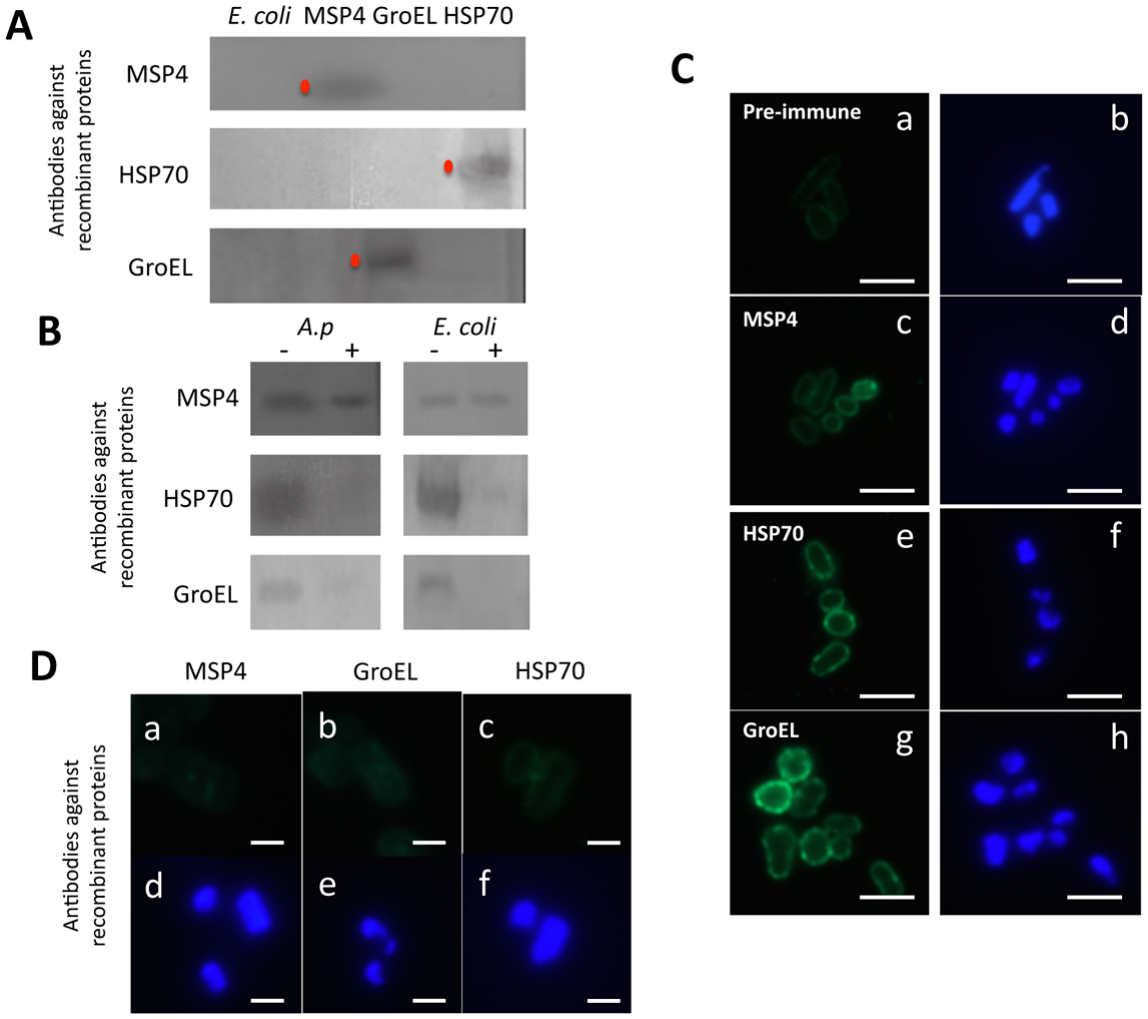
Specificity of antibodies produced in rabbits against *A*. *phagocytophilum* recombinant proteins. (A) Western blot analysis of 10 μg of recombinant *A*. *phagocytophilum* proteins produced in *E*. *coli* (red dots) demonstrates the specificity of the antibodies produced in rabbits. *E*. *coli* cell proteins were included as negative control. (B) *A*. *phagocytophilum* (NY18) purified from infected ISE6 tick cells and recombinant *E*. *coli* were mock treated (-) or surface digested with trypsin (+) and 10 μg protein loaded onto polyacrylamide gels for Western blot analysis using rabbit antibodies produced against recombinant proteins. (C) Immunofluorescence assay of *E*. *coli* producing recombinant *A*. *phagocytophilum* MSP4, HSP70 and GroEL proteins and reacted with (a) control pre-immune IgGs which gave similar results for all recombinant *E*. *coli* or purified antibodies against (c) MSP4, (e) HSP70, and (g) GroEL (green, FITC) or (b, d, f, h) stained with DAPI (blue). Bars, 10 μm. (D) Immunofluorescence assay of control *E*. *coli* producing recombinant tick Porin and reacted with purified antibodies against (a) MSP4, (b) GroEL, and (c) HSP70 (green, FITC) or (d-f) stained with DAPI to rule out cross-reaction of antibodies against *A*. *phagocytophilum* proteins with *E*. *coli* proteins. Bars, 10 μm. The *E*.*coli* induced for the production of recombinant *A*. *phagocytophilum* proteins were fixed with 4% paraformaldehyde and used for immunofluorescence. *E*. *coli* cells producing recombinant tick Porin were used as control.

Recombinant *A*. *phagocytophilum* GroEL, MSP4 and HSP70 proteins were produced in *Escherichia coli* to produce antibodies in rabbits. Antibodies against all recombinant proteins produced good results when used in Western blot and immunofluorescence analyses. The specificity of the antibodies was demonstrated by Western blot against *Anaplasma* and recombinant proteins (Fig 2A and 2B) (S1 Fig) and by immunofluorescence in which antibodies against recombinant *A*. *phagocytophilum* proteins recognized recombinant *E*. *coli* (Fig 2C) but did not react with *E*. *coli* cells expressing the recombinant tick mitochondrial Porin, thus ruling out cross-reaction with *E*. *coli* proteins (Fig 2D).

**Fig 3:**
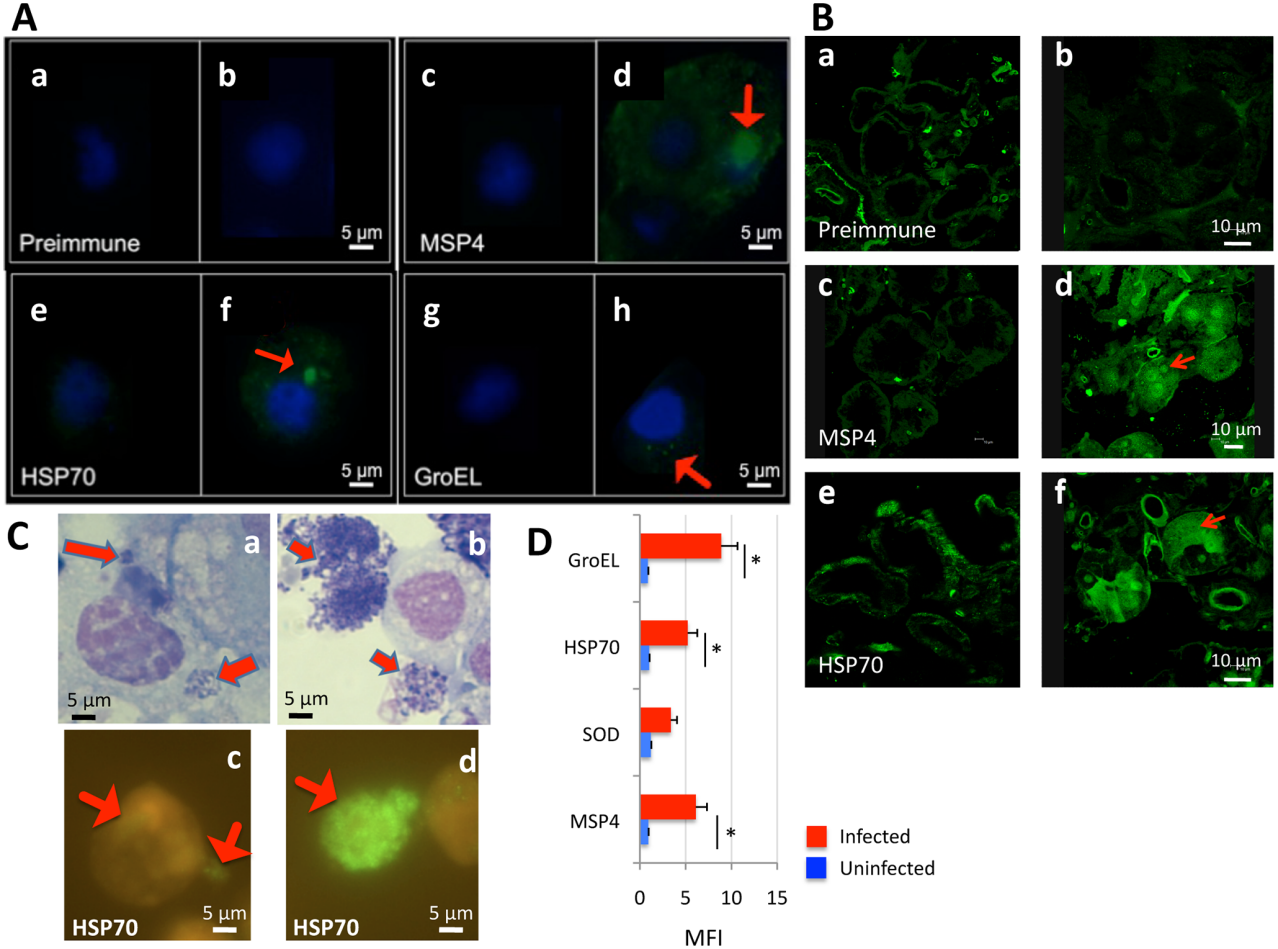
Antibodies against recombinant proteins recognize *A*. *phagocytophilum* in infected tick cells and ticks by immunofluorescence. (A) Uninfected and *A*. *phagocytophilum* (NY18)-infected ISE6 tick cells were characterized by immunofluorescence in (a, c, e, g) uninfected and (b, d, f, h) infected cells. Representative immunofluorescence images are shown for tick cells stained with rabbit preimmune (control) or anti-*A*. *phagocytophilum* protein antibodies (green, FITC; blue, DAPI). Arrows show the localization of *A*. *phagocytophilum* proteins in infected cells. Bars, 5 μm. (B) Sections were made from *I*. *scapularis* female ticks after feeding on an uninfected (a, c, e) or *A*. *phagocytophilum* (NY18)-infected (b, d, f) sheep. Representative immunofluorescence images are shown for salivary gland sections stained with rabbit preimmune (control) or anti-*A*. *phagocytophilum* protein antibodies (green, FITC). Arrows show the localization of *A*. *phagocytophilum* proteins in infected cells. Bars, 10 μm. (C) IDE8 tick cells were collected in low and high percentage *A*. *phagocytophilum* (L610)-infected cells and representative immunofluorescence images are shown. (a, b) Bright-field images of Giemsa-stained (a) low percentage and (b) high percentage infected tick cells. Bacteria stain purple (arrows) and host nuclei stain pink. (c) Low percentage and (d) high percentage infected tick cells were stained with rabbit anti-*A*. *phagocytophilum* HSP70 protein antibodies (green, FITC). Arrows show the localization of *A*. *phagocytophilum* proteins in infected cells. Bar, 5 μm. (D) Flow cytometry profile showing MFI values determined using a FITC-conjugated secondary antibody. *A*. *phagocytophilum* (NY18)-infected and uninfected control ISE6 tick cells were washed, fixed, permeabilized and incubated with primary unlabeled antibody (preimmune IgG isotype control, MSP4, SOD, HSP70 and GroEL), washed in PBS and incubated with FITC-goat anti-rabbit IgG. MFI was calculated as the MFI of the test-labeled sample minus the MFI of the isotype control, shown as Ave+SD and compared between infected and uninfected tick cells by Student's t-test (*P<0.05) (N = 3).

Antibodies against all recombinant proteins recognized *A*. *phagocytophilum* in high percentage infected *I*. *scapularis* ISE6 tick cells (Fig 3A) and female tick salivary glands (Fig 3B). However, GroEL was detected by immunofluorescence at very low levels in infected tick cells (Fig 3A and 3h) while MSP4 and HSP70 were clearly detected in bacterial morulae in infected cells (Fig 3A, 3d and 3f). To corroborate proteomics results, *A*. *phagocytophilum* HSP70 protein levels were characterized in low and high percentage infected tick cells by immunofluorescence. In infected IDE8 tick cells, *A*. *phagocytophilum* HSP70 levels increased as bacterial morulae matured and were present on many extracellular rickettsia after exiting the tick cell (Fig 3C), thus corroborating proteomics results for this protein. Flow cytometry was also used to characterize *A*. *phagocytophilum* MSP4, HSP70 and GroEL protein levels in high percentage infected and uninfected ISE6 tick cells and in comparison with superoxide dismutase (SOD) that was not over-represented in high percentage infected tick cells when compare to low percentage infected cells (Fig 3D). The results showed that MSP4, HSP70 and GroEL were recognized at higher levels in infected than in uninfected tick cells while for SOD differences were not statistically significant between samples, thus corroborating that MSP4, HSP70 and GroEL proteins were produced at higher levels in high percentage infected tick cells (Fig 3D).

The subcellular localization of selected *A*. *phagocytophilum* over-represented proteins was characterized in high percentage infected tick cells and salivary glands. Bacteria purified from infected ISE6 tick cells and recombinant *E*. *coli* were mock treated or surface digested with trypsin and proteins loaded onto polyacrylamide gels for Western blot analysis using rabbit antibodies produced against recombinant proteins. All recombinant proteins were sensitive to protease treatment after purification but the results showed that while MSP4 was resistant to trypsin digestion in both *A*. *phagocytophilum* and *E*. *coli*, HSP70 and GroEL were extracellular and exposed to protease digestion (Fig 2B). Furthermore, as previously shown for *A*. *marginale* major surface proteins [27], all three proteins were localized by immunofluorescence on the membrane of recombinant *E*. *coli* (Fig 2C). These results suggested that MSP4 may be a transmembrane protein and showed that HSP70 and GroEL are surface-exposed in *A*. *phagocytophilum* and recombinant *E*. *coli*.

### *A*. *phagocytophilum* over-represented and surface-exposed proteins are involved in rickettsia-tick interactions

GroEL, which belongs to the chaperonin/HSP60 family, prevents misfolding and promotes the refolding and proper assembly of unfolded polypeptides generated under stress conditions and has been used for genetic characterization of tick-borne bacteria [28]. HSP70 is also a chaperonin involved in protein folding and stress response [29]. Recently, GroEL and HSP70 were shown to relocate to the *Bacillus subtilis* membrane to restore membrane structure and function after ethanol stress [30] and to function in the molecular processing of *Borrelia burgdorferi* flagellin [31]. GroEL was also shown to function in *Caulobacter crescentus* multiplication and response to oxidative stress [32]. These results suggested that bacterial heat shock proteins function as molecular chaperones to protect cells from stress-induced lethal damage but also have important roles under physiological growth conditions by acting as carriers for immunogenic peptides, assisting in protein export or mediating adherence to host cells and may play an essential role during cell division [30–34]. The role of major surface proteins in adhesion to tick cells for bacterial infection has been demonstrated in *A*. *marginale* [24, 35–37] and MSP1a levels have been associated with bacterial differential adhesion to host cells [27, 38]. The GroEL and HSP70 proteins may be functionally relevant at the tick-*A*. *phagocytophilum* interface because as shown here they are localized on the cell membrane in *A*. *phagocytophilum* as in other bacteria [24, 30, 31, 33, 34, 39] and may interact with other membrane proteins [32].

**Fig 4:**
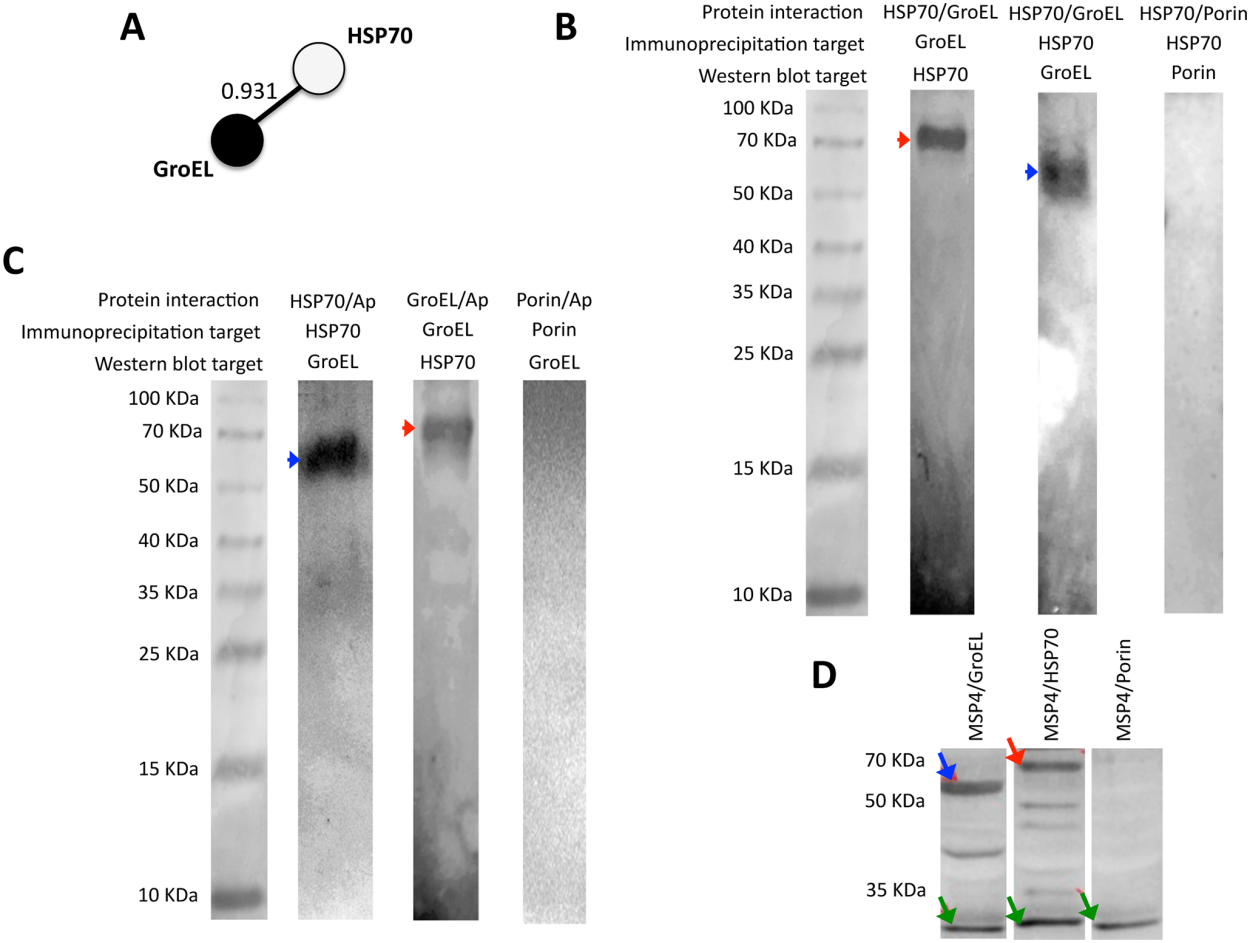
Characterization of *A*. *phagocytophilum* protein-protein interactions. (A) Protein-protein interactions were characterized *in silico* using STRING 8.3 (http://string-db.org). The STRING score value is shown, defined as threshold of significance to include the interaction (maximum value = 1) computed by combining the probabilities from the different evidence channels, correcting for the probability of randomly observing an interaction. (B) Protein-protein interactions were characterized *in vitro* using *A*. *phagocytophilum* HSP70 (red arrow) and GroEL (blue arrow) recombinant proteins and tick Porin as control [15]. The proteins were mixed in equimolar amounts and immunoprecipitated using anti-GroEL or anti-HSP70 antibodies and Protein G Dynabeads. The purified proteins were eluted using Laemmli sample buffer and loaded onto a 12% SDS-PAGE gel for Western blot analysis using anti-HSP70, anti-GroEL or anti-Porin antibodies. (C) Protein-protein interactions were characterized *in vitro* using *A*. *phagocytophilum* protein extracts, recombinant HSP70 (red arrow) and GroEL (blue arrow) proteins and tick Porin as control [15]. Protein G Dynabeads were incubated with purified anti-HSP70, anti-GroEL or anti-Porin antibodies and then 130 μg of *A*. *phagocytophilum* proteins were added. Unbound proteins were removed and the beads were washed three times with PBS with addition of 0.1% Triton X-100, resuspended in Laemmli sample buffer and loaded onto a 12% SDS-PAGE gel for Western blot analysis using anti-HSP70 or anti-GroEL antibodies. (D) Protein-protein interactions were characterized *in vitro* using *A*. *phagocytophilum* HSP70 (red arrow), GroEL (blue arrow) and MSP4 (green arrows) recombinant proteins and tick Porin as control [15]. Nickel beads were covered with histidine-tagged MSP4, washed and incubated with GroEL or HSP70, MSP4 or Porin as control. After incubation, beads were washed and proteins eluted in Laemmli sample buffer and loaded onto a 15% SDS-PAGE gel.

The characterization of protein-protein direct and/or functional interactions using STRING suggested that GroEL possibly interacts with HSP70 (Fig 4A), suggesting a physical and/or functional connection between these proteins. These proteins have been shown to interact directly (physically) or indirectly (functionally) in other tick-borne bacteria but data were not available for *A*. *phagocytophilum* [31]. The interaction between recombinant *A*. *phagocytophilum* HSP70 and GroEL proteins was confirmed using immunoprecipiation where GroEL was co-purified with HSP70 using anti-HSP70 or anti-GroEL antibodies (Fig 4B). Furthermore, the interaction between recombinant proteins with *A*. *phagocytophilum* protein extracts corroborated the interaction between GroEL and HSP70 after immunoprecipitation and Western blot analysis (Fig 4C). The interaction between MSP4 and GroEL or HSP70 was confirmed using histidine-tagged MSP4 and nickel beads (Fig 4D).

**Fig 5:**
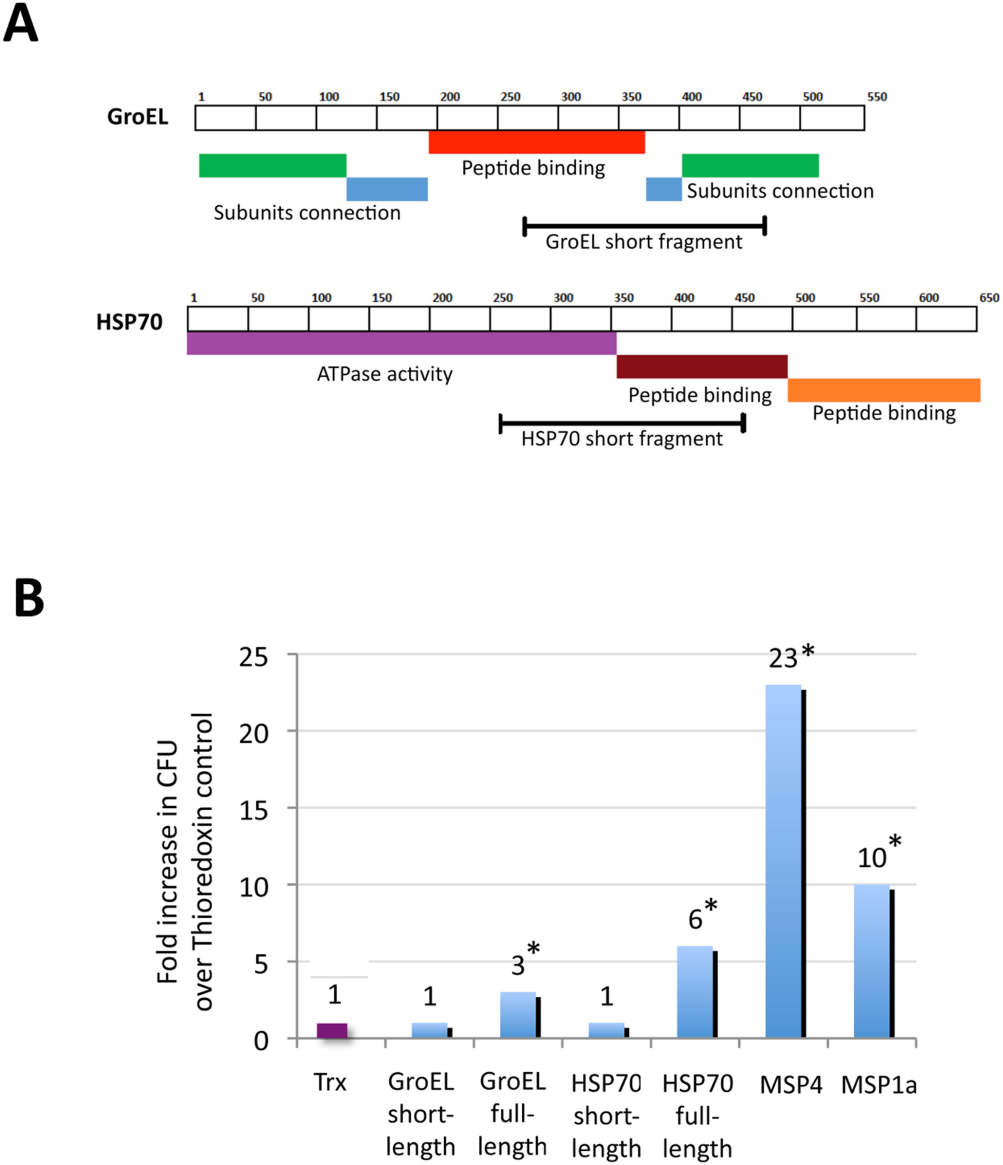
Characterization of *A*. *phagocytophilum* protein adhesion to tick cells. (A) Schematic representation of GroEL and HSP70 functional domains in full-length and short-length proteins produced in *E*. *coli* and used for binding experiments to tick cells. (B) *E*. *coli* strains were grown and induced for the production of recombinant proteins. *E*. *coli* strains with expression vector alone producing recombinant Thioredoxin and *A*. *marginale* MSP1a were used as negative and positive control, respectively. Adhesive bacteria were quantitated as the number of colony forming units (CFU) recovered from each test, shown as fold increase and compared to the Thioredoxin (Trx) control values by Student’s t-test for paired samples (*P<0.05; Table 1).

To characterize the possible role of these proteins in *Anaplasma*-tick interactions, the binding of GroEL, HSP70 and MSP4 to tick cells was assessed using the recombinant *E*. *coli* model [27] in which *E*. *coli* producing surface-exposed *A*. *phagocytophilum* proteins were used for binding to tick cells (Table 1). The results demonstrated that GroEL, HSP70 and MSP4 were involved in binding to tick cells (Table 1). Furthermore, *E*. *coli* producing recombinant GroEL and HSP70 with truncated peptide-binding domains that are involved in protein-protein interactions (Fig 5A) did not bind to tick cells, providing additional support for the role of these proteins in *A*. *phagocytophilum*-tick interactions (Fig 5B). Remarkably, binding to tick cells was more pronounced in bacteria producing recombinant *A*. *phagocytophilum* MSP4 than the *A*. *marginale* MSP1a positive control [27, 38] (Fig 5B).

**Table 1:** Adhesion to cultured tick cells by recombinant *E*. *coli* producing surface-exposed *A*. *phagocytophilum* proteins.

Protein	No. CFU (mean ± SE) recovered from ISE6 tick cells (number of replicates)
Thioredoxin (expression vector alone)–Negative control	7±3 (N = 6)
*A*. *phagocytophilum* GroEL short-length	6±1 (N = 2)
*A*. *phagocytophilum* GroEL full-length	17±6 (N = 4)*
*A*. *phagocytophilum* HSP70 short-length	5±2 (N = 2)
*A*. *phagocytophilum* HSP70 full-length	41±28 (N = 4)*
*A*. *phagocytophilum* MSP4	152±50 (N = 4)*
*A*. *marginale* MSP1a—Positive control	65±20 (N = 4)*

*E*. *coli* strains were grown and induced for the production of recombinant proteins. *E*. *coli* strains with expression vector alone producing recombinant Thioredoxin and *A*. *marginale* MSP1a were used as negative and positive control, respectively. Adhesive bacteria were quantitated as the number of colony forming units (CFU) recovered from each test and compared to the Thioredoxin control values by Student’s t-test for paired samples (*P<0.05). GroEL and HSP70 were produced as full-length and short-length (amino acids 275–475 and 262–460 for GroEL and HSP70, respectively) proteins.

**Fig 6:**
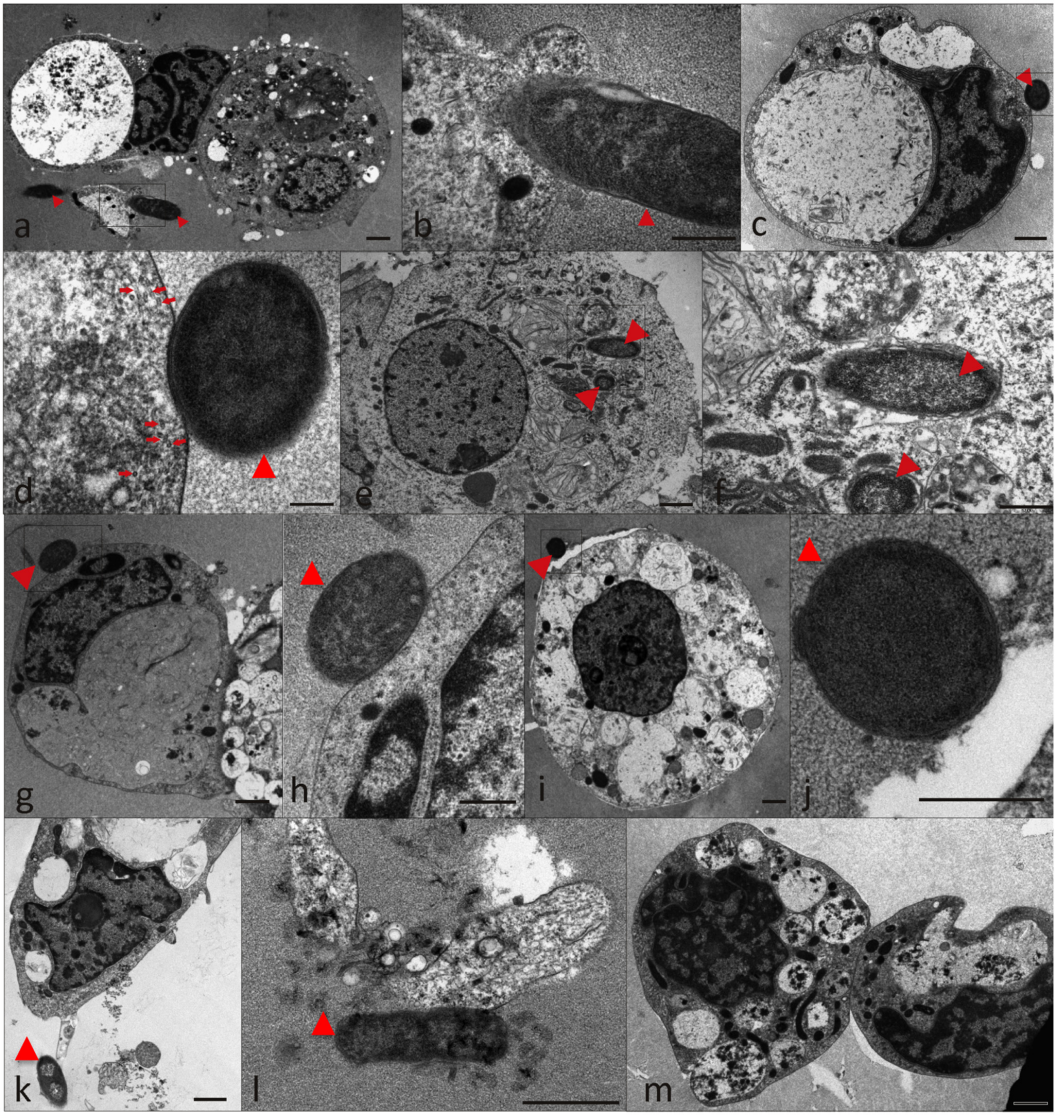
Electron micrographs of ISE6 tick cells reacted with recombinant *E*. *coli* producing *A*. *phagocytophilum* recombinant proteins. Representative images of recombinant *E*. *coli* shown by arrowheads adhered to tick cells. (a, b) *A*. *marginale* MSP1a positive control; (c-f) MSP4; (g-k) HSP70; (l) GroEL; (m) Thioredoxin negative control. Microtubules are shown by arrows in (d). Scale bars, 1 μm (a, c, e, g, i, k-m), 500 nm (b, d, f, h, j).

The interaction of recombinant *E*. *coli* producing *A*. *marginale* MSP1a (positive control; Fig 6a and 6b) and *A*. *phagocytophilum* MSP4 (Fig 6c–6f), HSP70 (Fig 6g–6k) and GroEL (Fig 6l) proteins was also characterized by electron microscopy in comparison to Thioredoxin-producing cells (Fig 6m) to provide additional evidence for the role of these proteins in rickettsia-tick interactions. The results showed not only interaction with tick cells for bacteria with surface exposed *A*. *phagocytophilum* MSP4, HSP70 and GroEL (Fig 6c–6l) but also internalization of recombinant bacteria producing MSP4 (Fig 6e and 6f). Bacterial internalization was observed for MSP1a and MSP4 only (Fig 6e and 6f) and the presence of microtubules close to tick cell plasma membrane suggested the beginning of the process of phagosome formation (Fig 6d). An insight into the three-dimensional cell morphology during the interaction between MSP4-producing *E*. *coli* and tick cells revealed the presence of small vesicles inside a tick cell phagocytic cup (S1 Video). Interestingly, one vesicle was in direct contact with the bacterial outer membrane suggesting a bacterial origin for these vesicles (S1 Video). These small vesicles were clearly distinguished only by electron tomography (S1 Video) and measured from 12 to 34 nm in diameter (mean value ± SD = 20.4 ± 5.5 nm; N = 37).

**Fig 7:**
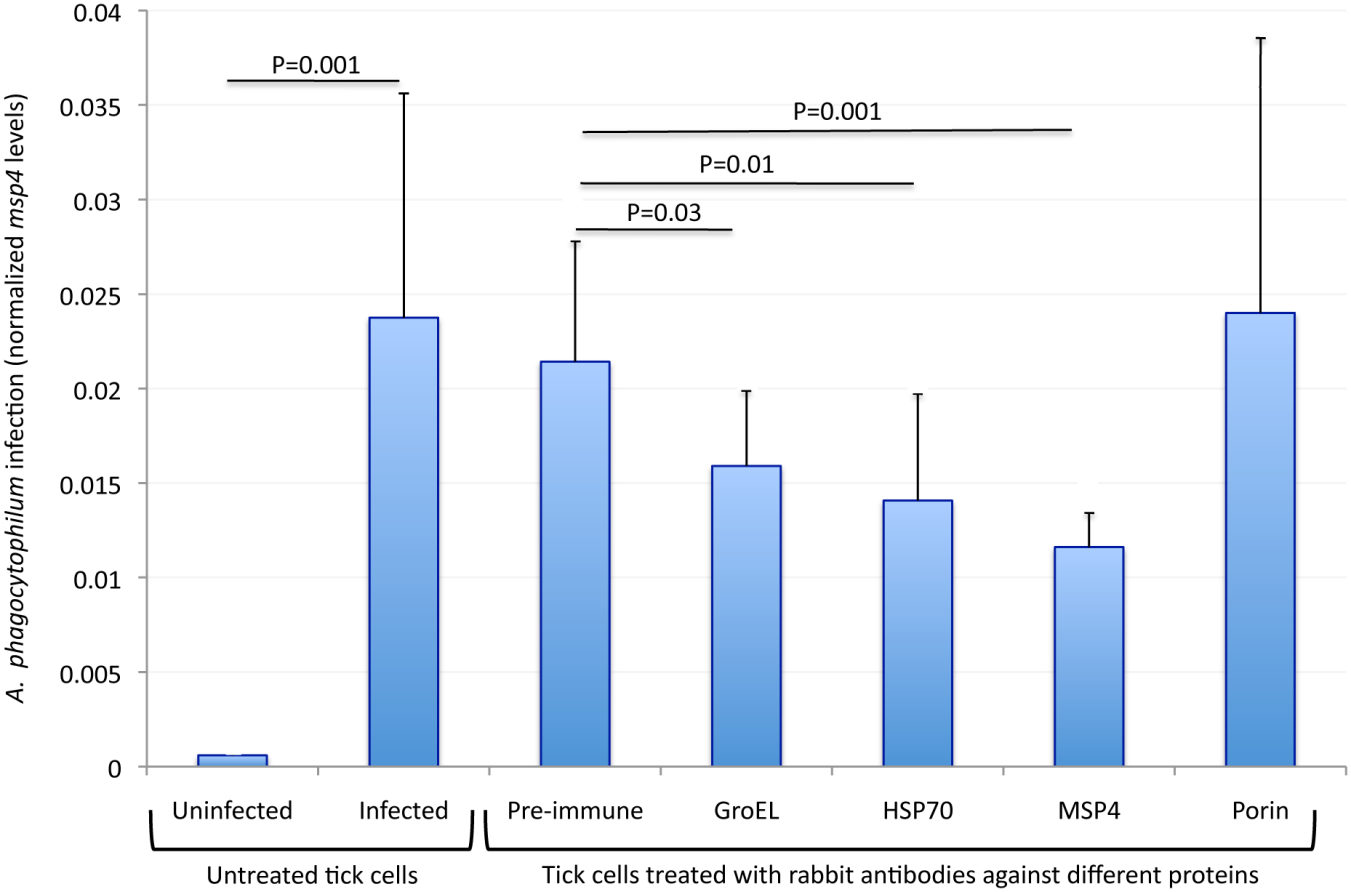
Inhibition of *A*. *phagocytophilum* infection by antibodies against over-represented proteins. The antibodies against surface-exposed proteins, GroEL, HSP70 and MSP4, were used to characterize the inhibition of pathogen infection in ISE6 tick cells. Tick cells were treated with different rabbit antibodies and then infected with *A*. *phagocytophilum* (NY18). Treatments included rabbit pre-immune serum, anti-*A*. *phagocytophilum* GroEL, HSP70 and MSP4 protein antibodies and anti-tick Porin antibodies. Untreated cells were left uninfected or infected with *A*. *phagocytophilum* (NY18). *A*. *phagocytophilum* infection levels were determined by *16S rDNA* and *msp4* PCR and normalized against tick *16S* mitochondrial *rDNA* with similar results. Normalized *msp4* levels are shown in arbitrary units as Ave+S.D and were compared between infected and uninfected untreated cells and between infected cells treated with the pre-immune serum and antigen-specific antibodies by Student’s t-test with unequal variance (P<0.05; N = 4 replicates per treatment).

To provide additional support for the role of *A*. *phagocytophilum* GroEL, HSP70 and MSP4 proteins in rickettsia-tick interactions, antibodies against these proteins were used to inhibit infection of tick cells. Antibodies against these proteins could affect the interaction between bacterial ligands and tick receptors to block infection or affect the interaction with proteins functionally important for bacterial infection and/or multiplication in tick cells. The results showed an inhibitory effect of anti-GroEL, anti-HSP70 and anti-MSP4 but not anti-tick Porin antibodies on *A*. *phagocytophilum* infection of tick cells when compared to cells treated with pre-immune serum (Fig 7), thus corroborating the role of these proteins in rickettsial adhesion/invasion of tick cells.

It has been shown that bacterial GroEL interacts with lectin-like oxidized low-density lipoprotein receptor-1 (LOX-1) to mediate bacterial adherence to vertebrate host cells [34]. However, the LOX-1 homolog was not identified in the *I*. *scapularis* genome, thus suggesting that the adhesion of *A*. *phagocytophilum* to tick cells may not be through interactions between GroEL and LOX-1.

The increase in protein levels in high percentage infected tick cells and tick salivary glands (S1 and S2 Tables) and the inhibition of bacterial infection in tick cells treated with protein-specific antibodies (Fig 7) supported the hypothesis that higher GroEL, HSP70 and MSP4 levels facilitate *A*. *phagocytophilum* infection of tick cells through interaction with a still unknown receptor or interacting molecule. The origin of the vesicles observed in tick cells interacting with recombinant *E*. *coli* with membrane-exposed MSP4 in the process of phagosome formation is unknown. However, it can be hypothesized that the vesicles arising from the outer membrane of MSP4-expressing *E*. *coli* were released as a response to phagocytosis. As described in other Gram-negative bacteria, the production of these possible outer membrane vesicles can be stress-induced and/or involved in pathogenesis through secretion of virulence factors, adhesins and degradative enzymes [40–42]. Additionally, protein-protein interactions between HSP70, GroEL and MSP4 suggested that these proteins might form a complex on the rickettsial membrane to facilitate the interaction with tick cells.

### The *A*. *phagocytophilum* type IV secretion system may be involved in the secretion of stress response proteins during rickettsial infection and multiplication in ticks

The mechanism by which *A*. *phagocytophilum* HSP70 and GroEL are processed to appear as surface-exposed proteins is unknown. As other Gram-negative bacteria [43], *A*. *phagocytophilum* has a type IV secretion system (T4SS) that translocates effector molecules to host cells to exert their activity on transcription and apoptosis and favor rickettsial infection [44–46]. These effector molecules have not been fully characterized but may be responsible for some of the changes occurring in tick transcriptome and proteome in response to rickettsial infection. Herein, we speculate that some of the *A*. *phagocytophilum* stress response proteins over-represented during rickettsial infection may constitute T4SS effectors.

Preliminary results showed that *A*. *phagocytophilum* VirB2, VirB6, VirB10 and VirD4 were over-represented in tick salivary glands (S2A Fig), and their homologs played a central role in the structure and function of the *E*. *coli* T4SS [43]. Additionally, the preliminary analysis of *A*. *phagocytophilum* proteins present in tick salivary glands and/or high percentage infected tick cells using the S4TE software [47] to identify candidate T4SS effectors suggested that Iron-binding protein (APH_0051), Peptidase (APH_1159) and HSP70 (APH_0346) harbor several characteristic features of proteins that may be secreted by the T4SS (S2B Fig). S4TE is an easy-to-use and customizable algorithm that do not use a machine learning approach for the prediction of candidate effector proteins secreted by T4SS in genomes of any size [47]. Even though further functional validation is needed to confirm this hypothesis, the *A*. *phagocytophilum* T4SS may be involved in the secretion of stress response proteins such as HSP70 during rickettsial infection and multiplication in ticks.

### *A*. *phagocytophilum* over-represented proteins are regulated at the transcriptional level

**Fig 8:**
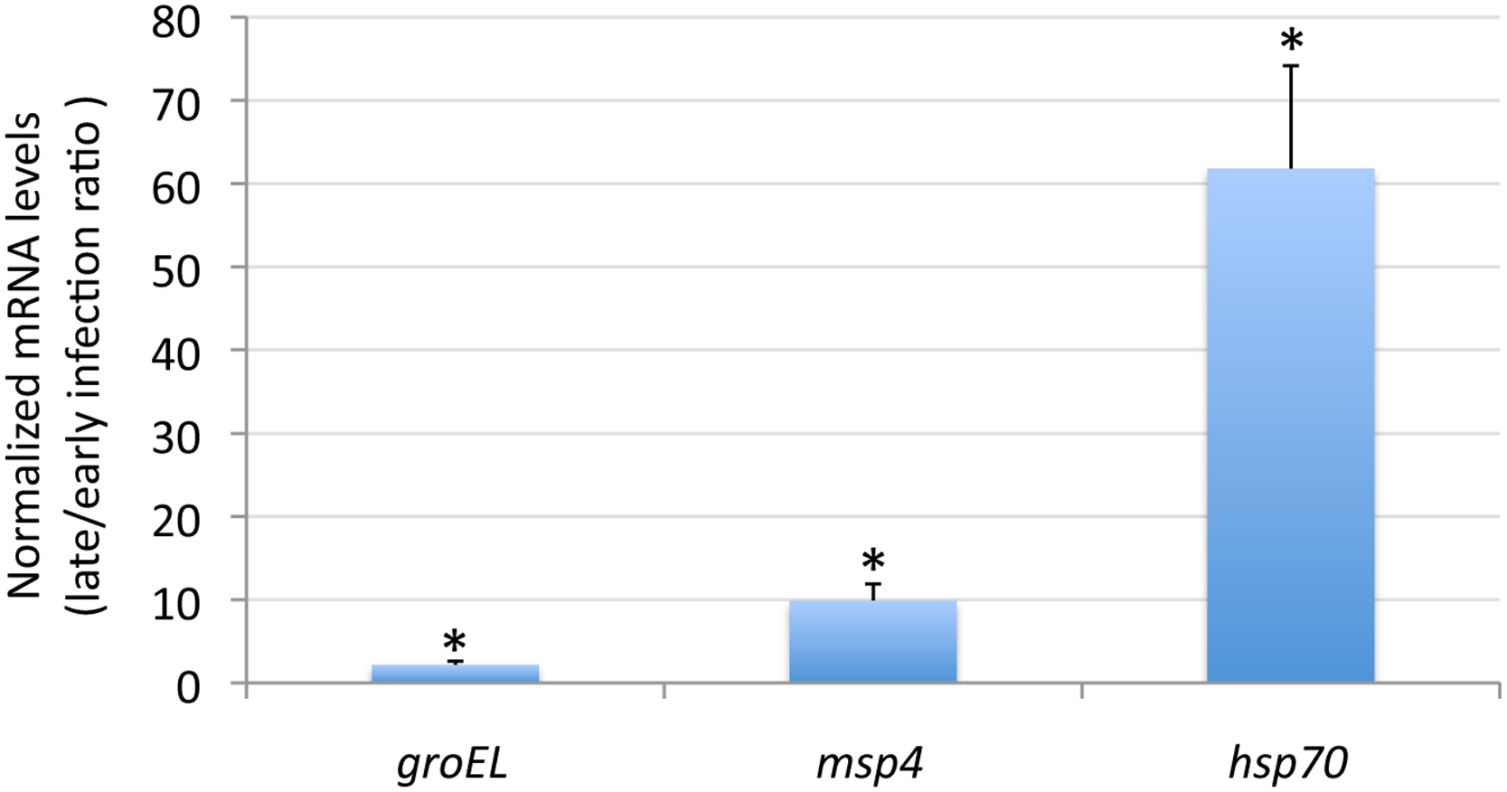
Characterization of the mRNA levels for selected genes encoding for *A*. *phagocytophilum* over-represented proteins. The mRNA levels for *groEL*, *msp4* and *hsp70* were determined by real-time RT-PCR in low and high percentage infected ISE6 tick cells. Amplification efficiencies were normalized against tick *16S rRNA* and mRNA levels expressed in arbitrary units. The ratio of normalized mRNA levels in high to low percentage-infected cells was represented as Ave+SD. Normalized Ct values were compared between low and high percentage infected tick cells by Student's t-test (*P<0.05) (N = 5).

The mRNA levels were characterized in low and high percentage infected tick cells for *A*. *phagocytophilum groEL*, *msp4* and *hsp70* encoding proteins over-represented in high percentage infected tick cells and salivary glands when compared to low percentage infected tick cells and guts, respectively. The results of the RT-PCR demonstrated that mRNA levels were significantly higher in high percentage infected cells (Fig 8). These results suggested that *A*. *phagocytophilum* GroEL, MSP4 and HSP70 over-represented proteins in high percentage infected cells are regulated at the transcriptional level. However, as described in other bacteria, post-transcriptional mechanisms such as enhancement of mRNA stability and/or translation [48] may also affect GroEL, MSP4 and HSP70 protein levels. In *A*. *phagocytophilum*, the activation of *p44* has been linked to post-transcriptional bacterial RNA splicing [49] which may account for the increase in protein levels observed in high percentage infected tick cells.

## Conclusions

**Fig 9:**
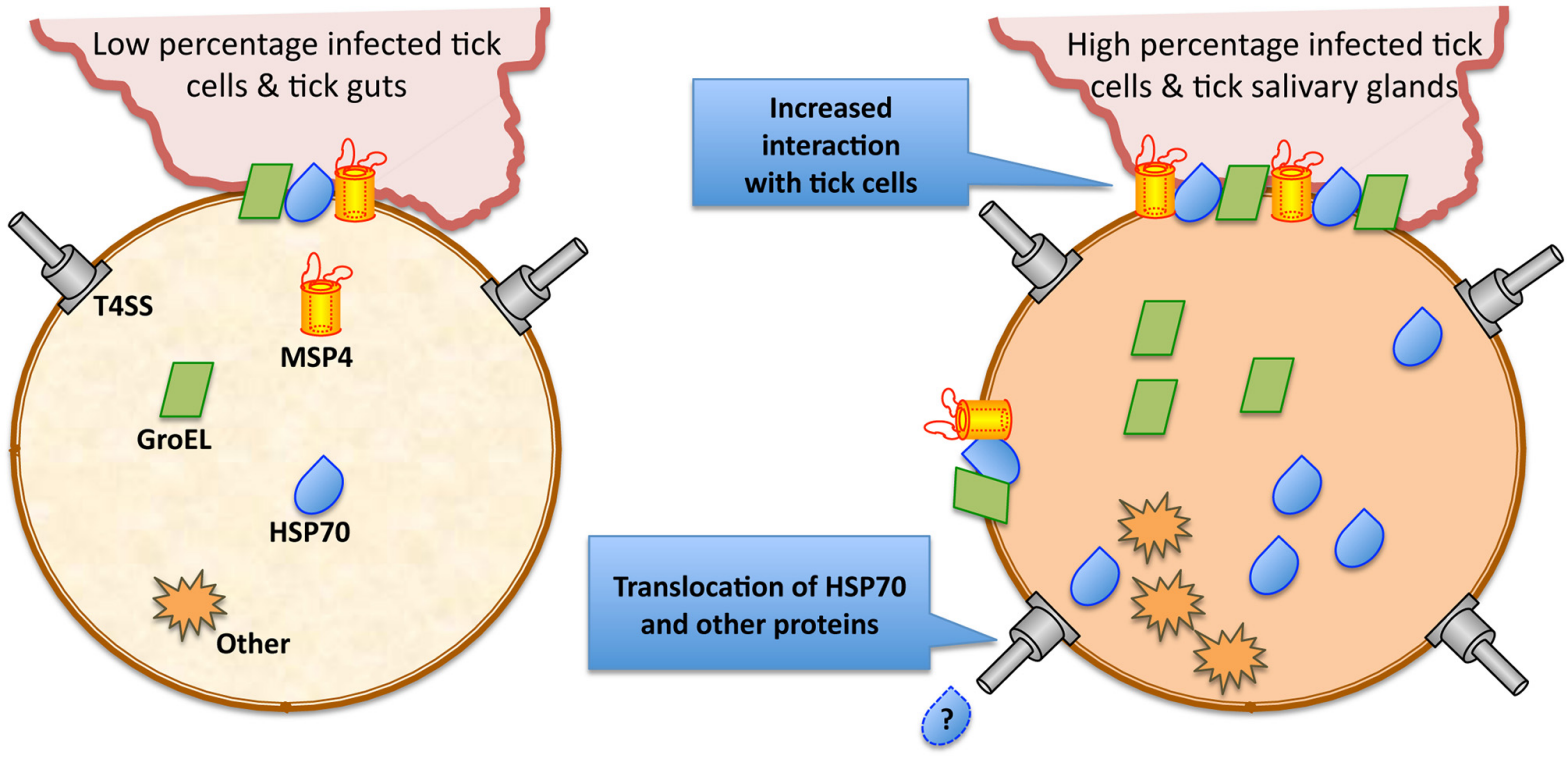
Proposed mechanisms of how stress response and surface proteins facilitate *A*. *phagocytophilum* infection in high percentage infected tick cells and tick salivary glands. The levels of certain bacterial stress response and surface proteins are higher in high percentage *A*. *phagocytophilum*-infected tick cells and tick salivary glands. MSP4, GroEL and HSP70 interact and bind to tick cells, thus facilitating rickettsia-tick interactions and infection. In high percentage infected tick cells and tick salivary glands, bacteria reduce multiplication once they infect the cells but infection is required to complete the life cycle and get ready for transmission. The activation of stress response proteins in *A*. *phagocytophilum* may represent a mechanism by which rickettsiae increase infection by facilitating interaction with tick cells and protecting bacteria against stress. The T4SS may be associated with the secretion of HSP70 and other stress response proteins. Abbreviations: T4SS, Type IV Secretion System; Question mark indicates that secretion of HSP70 in a T4SS-dependent manner remains to be proved.

In this study we have characterized *A*. *phagocytophilum* proteome during rickettsial multiplication in *I*. *scapularis* cultured tick cells, guts and salivary glands and provided new evidences on the behavior of this pathogen in ticks. Cultured tick cells are a good model for the study of tick-pathogen interactions and the tick tissues selected for this study play a major role during pathogen acquisition, multiplication and transmission. The results of these studies showed that in agreement with previous findings at the mRNA and protein levels [21], the proteome of *A*. *phagocytophilum* in tick salivary glands had a higher impact on protein synthesis and processing than on bacterial replication. These results correlated well with the developmental cycle of *A*. *phagocytophilum*, in which rickettsia convert from an intracellular reticulated, replicative form to the nondividing infectious dense-core form. Additionally, some of the proteins identified here were predicted based on genomics information [50] and proteomics results corroborated the existence of these proteins, thus expanding linkage of the genome annotation with the proteome. The most important finding of these studies was the increase in the level of bacterial stress response and surface proteins in high percentage *A*. *phagocytophilum*-infected tick cells and salivary glands with implications for tick-pathogen interactions (Fig 9). These results supported our hypothesis that *A*. *phagocytophilum* proteins that increase as infection proceeds in cultured tick cells and ticks are important for pathogen infection and gave a new dimension to the role of certain stress response and surface proteins during *A*. *phagocytophilum* infection in ticks. These results also demonstrated that MSP4, GroEL and HSP70 could interact and bind to tick cells, thus playing a role in rickettsia-tick interactions (Fig 9). The MSP4 interaction with tick cells may induce the secretion of vesicles at the phagocytic cup to aid in adhesin secretion for rickettsial infection. The T4SS may be involved in the secretion of some stress response proteins (Fig 9). As suggested previously for *A*. *marginale*, these proteins may be targeted as antigens for use in vaccine development for control of *Anaplasma* infection in tick vectors by reducing or blocking transmission to vertebrate hosts [37]. Additionally, these proteins could be used to develop novel therapeutic interventions for *A*. *phagocytophilum* and future experiments should evaluate these proteins as antigens for vaccination against HGA.

## Methods

### *I*. *scapularis* tick cells and sample preparation

The *I*. *scapularis* embryo-derived tick cell line ISE6, provided by Ulrike Munderloh, University of Minnesota, USA, was cultured in L15B300 medium as described previously [37], except that the osmotic pressure was lowered by the addition of one-fourth sterile water by volume. The ISE6 cells were first inoculated with *A*. *phagocytophilum* (human NY18 isolate)-infected HL-60 cells [51] and maintained according to Munderloh et al. [52] until infection was established and routinely passaged. Infected ISE6 cells were frozen in liquid nitrogen and served as inoculum for uninfected cells. Uninfected and infected cultures (N = 3 independent cultures each) were sampled at 3 days post-infection (dpi) (low percentage infected cells, 10–15% (Ave±SD, 12±2)) and 8 dpi (high percentage infected cells, 65–71% (Ave±SD, 68±3)). The percentage of cells infected with *A*. *phagocytophilum* was calculated by examining at least 200 cells using a 100x oil immersion objective. The cells were centrifuged at 10,000 g for 3 min, and cell pellets were frozen in liquid nitrogen until used for protein and RNA extraction. Approximately 10^7^ cells were pooled from each condition and homogenized with a needle (27G) in 500 μl lysis buffer (phosphate buffered saline (PBS), 1% Triton X-100, supplemented with Complete protease inhibitor cocktail (Roche, Basel, Switzerland). Samples were sonicated for 1 min in an ultrasonic cooled bath followed by 10 sec of vortexing. After 3 cycles of sonication-vortexing, total cell extracts were centrifuged at 200 x g for 5 min to remove cell debris. The supernatants were collected and protein concentration was determined using the Bradford Protein Assay (Bio-Rad, Hercules, CA, USA) with BSA as standard. Total RNA was extracted from the same cell cultures (N = 5) using TriReagent (Sigma, St. Louis, MO, USA) following manufacturer’s recommendations.

### *I*. *scapularis* ticks and sample preparation

*I*. *scapularis* ticks were obtained from the laboratory colony maintained at the Oklahoma State University Tick Rearing Facility. Larvae and nymphs were fed on rabbits and adults were fed on sheep. Off-host ticks were maintained in a 12 hr light: 12 hr dark photoperiod at 22–25°C and 95% relative humidity. Adult female *I*. *scapularis* were infected with *A*. *phagocytophilum* (NY18) by feeding on a sheep inoculated intravenously with approximately 1x10^7^ *A*. *phagocytophilum*-infected HL-60 cells (90–100% infected cells) [53]. In this model, over 85% of ticks become infected with *A*. *phagocytophilum* in guts and salivary glands [53]. One hundred female adult ticks were removed from the sheep 7 days after infestation, held in the humidity chamber for 4 days and dissected for protein extraction from guts and salivary glands. Uninfected ticks were prepared in a similar way but feeding on an uninfected sheep. Two independent samples were collected and processed for each tick tissue. Total proteins were extracted from uninfected and *A*. *phagocytophilum*-infected gut and salivary gland samples using the AllPrep DNA/RNA/Protein Mini Kit (Qiagen, Valencia, CA, USA). Animals were housed and experiments conducted with the approval and supervision of the Oklahoma State University Institutional Animal Care and Use Committee (Animal Care and Use Protocol, ACUP No. VM1026).

### Proteomics data collection and analysis for *A*. *phagocytophilum* proteome in ISE6 tick cells

Protein extracts (150 μg) from the four experimental conditions, low percentage infected tick cells and matching uninfected control at 3 dpi and high percentage infected tick cells and matching uninfected control at 8 dpi were on-gel concentrated by SDS-PAGE and trypsin digested as described previously [54]. The desalted protein digest was resuspended in 0.1% formic acid and analyzed by RP-LC-MS/MS using an Easy-nLC II system coupled to an ion trap LTQ mass spectrometer (Thermo Scientific). The peptides were concentrated (on-line) by reverse phase chromatography using a 0.1×20 mm C18 RP precolumn (Thermo Scientific), and then separated using a 0.075×100 mm C18 RP column (Thermo Scientific) operating at 0.3 ml/min. Peptides were eluted using a 180-min gradient from 5 to 40% solvent B (Solvent A: 0,1% formic acid in water, solvent B: 0,1% formic acid in acetonitrile). ESI ionization was done using a Fused-silica PicoTip Emitter ID 10 mm (New Objective, Woburn, MA, USA) interface. Peptides were detected in survey scans from 400 to 1600 amu (1 mscan), followed by fifteen data dependent MS/MS scans (Top 15), using an isolation width of 2 mass-to-charge ratio units, normalized collision energy of 35%, and dynamic exclusion applied during 30 sec periods.

The MS/MS raw files were searched against a compiled database containing all sequences from Ixodida (77,195 Uniprot entries in March 2015) and Anaplasmataceae (64,677 Uniprot entries in March 2015) (http://www.uniprot.org) using the SEQUEST algorithm (Proteome Discoverer 1.4, Thermo Scientific). The following constraints were used for the searches: tryptic cleavage after Arg and Lys, up to two missed cleavage sites, and tolerances of 1 Da for precursor ions and 0.8 Da for MS/MS fragment ions and the searches were performed allowing optional Met oxidation and Cys carbamidomethylation.

A false discovery rate (FDR) < 0.05 was considered as condition for successful peptide assignments and at least 2 peptides per protein were the necessary condition for protein identification (S1 Table). After discarding tick proteins, proteins with the same description in the Anaplasmataceae were grouped and the total number of PSM for each protein were normalized against the total number of PSM on each infected tick cells and compared between low and high percentage infected tick cells by Chi2-test (P = 0.05) to select over-represented proteins in high percentage infected cells when compared to low percentage infected cells. Results are the mean of three replicates (S1 Table). Protein ontology analysis for BP was done using the STRAP software (Software for Researching Annotations of Proteins; [http://www.bumc.bu.edu/cardiovascularproteomics/cpctools/strap/] developed at the Cardiovascular Proteomics Center of Boston University School of Medicine (Boston, MA, USA). Protein ontology annotations were done with a probability cutoff value of 0.5 for importing protXML files and using the UniProtKB database.

### Proteomics data collection and analysis for *A*. *phagocytophilum* proteome in adult female tick guts and salivary glands

Proteins were digested using the filter aided sample preparation (FASP) protocol [55]. Briefly, samples were dissolved in 50 mM Tris-HCl pH8.5, 4% SDS and 50 mM DTT, boiled for 10 min and centrifuged. Protein concentration in the supernatant was measured by the Direct Detect system (Millipore, Billerica, MA, USA). About 150 μg of protein were diluted in 8 M urea in 0.1 M Tris-HCl (pH 8.5) (UA), and loaded onto 30 kDa centrifugal filter devices (FASP Protein Digestion Kit, Expedeon, TN, USA). The denaturation buffer was replaced by washing three times with UA. Proteins were later alkylated using 50 mM iodoacetamide in UA for 20 min in the dark, and the excess of alkylation reagents were eliminated by washing three times with UA and three additional times with 50 mM ammonium bicarbonate. Proteins were digested overnight at 37°C with modified trypsin (Promega, Madison, WI, USA) in 50 mM ammonium bicarbonate at 40:1 protein:trypsin (w/w) ratio. The resulting peptides were eluted by centrifugation with 50 mM ammonium bicarbonate (twice) and 0.5 M sodium chloride. Trifluoroacetic acid (TFA) was added to a final concentration of 1% and the peptides were finally desalted onto C18 Oasis-HLB cartridges and analyzing by LC-MS/MS using a C-18 reversed phase nano-column (75 μm I.D. x 50 cm, 3 μm particle size, Acclaim PepMap 100 C18; Thermo Fisher Scientific, Waltham, MA, USA) in a continuous acetonitrile gradient consisting of 0–30% B in 145 min, 30–43% B in 5 min and 43–90% B in 1 min (A = 0.5% formic acid; B = 90% acetonitrile, 0.5% formic acid). A flow rate of ca. 300 nl/min was used to elute peptides from the reverse phase nano-column to an emitter nanospray needle for real time ionization and peptide fragmentation on orbital ion trap mass spectrometer model Orbitrap Elite (Thermo Fisher Scientific).

The MS/MS raw files were searched as described previously for the ISE6 tick cells allowing, in this case, precursor and fragment mass tolerances of 600 ppm and 1200 mmu, respectively. Peptide identification was validated using the probability ratio method [56] and false discovery rate (FDR) was calculated using inverted databases and the refined method [57] with an additional filtering for precursor mass tolerance of 12 ppm. A false discovery rate (FDR) < 0.05 was considered as condition for successful peptide assignments and at least 2 peptides per protein were the necessary condition for protein identification (S2 Table). Proteins with the same description in the Anaplasmataceae were grouped and the total number of PSM for each protein were normalized against the total number of PSM on each infected adult female tick tissue and compared between salivary glands and guts by Chi2-test (P = 0.05) to select over-represented proteins in salivary glands when compared to tick guts. Results are the mean of two replicates. This experimental approach was previously used for the characterization of the tick proteome in response to *A*. *phagocytophilum* infection [58]. Protein ontology analysis for BP was done using the STRAP software (Software for Researching Annotations of Proteins; [http://www.bumc.bu.edu/cardiovascularproteomics/cpctools/strap/] developed at the Cardiovascular Proteomics Center of Boston University School of Medicine (Boston, MA, USA). Protein ontology annotations were done with a probability cutoff value of 0.5 for importing protXML files and using the UniProtKB database.

### Production of recombinant proteins and antibody preparation

The recombinant *A*. *phagocytophilum* (NY18) proteins GroEL, MSP4 (AFD54597), HSP70, and SOD were produced in *E*. *coli* BL21 cells (Champion pET101 Directional TOPO Expression kit, Carlsbad, CA, USA), induced with IPTG and purified using the Ni-NTA affinity column chromatography system (Qiagen Inc., Valencia, CA, USA) following manufacturer’s recommendations. Tick mitochondrial Porin was obtained as previously described [15]. Purified proteins were used to immunize rabbits and IgGs from preimmune and immunized animals were purified (Montage Antibody Purification Kit and Spin Columns with PROSEP-A Media, Millipore, Billerica, MA, USA) and used for analysis.

### Flow cytometry of tick cells incubated with antibodies

*A*. *phagocytophilum* (NY18)-infected and uninfected control ISE6 tick cells were washed in phosphate buffered saline (PBS), fixed and permeabilized with Intracell fixation and permeabilization kit (Inmunostep, Salamanca, Spain) following manufacturer recommendations. After permeabilization, the cells were washed in PBS and incubated with primary unlabeled antibody (preimmune IgG isotype control, MSP4, SOD, HSP70 and GroEL; 50 μg ml^-1^), washed in PBS and incubated in 100 μl of PBS with FITC-goat anti-rabbit IgG (Sigma, Madrid, Spain) labeled antibody (diluted 1/500) for 15 min at 4°C. Finally, the cells were washed with PBS and resuspended in 500 μl of PBS. All samples were analyzed on a FACScalibur Flow Cytometer, equipped with the CellQuest Pro software (BD-Biosciences, Madrid, Spain). The viable cell population was gated according to forward scatter and side scatter parameters. The level of MSP4, SOD, HSP70 and GroEL in the viable *A*. *phagocytophilum*-infected and uninfected tick cells was determined as the geometric median fluorescence intensity (MFI) of the test-labeled sample minus the MFI of the isotype control [15] and compared between infected and uninfected cells by Student's t-test (P = 0.05).

### Immunofluorescence in cultured tick cells

Antibodies against *A*. *phagocytophilum* GroEL, MSP4 and HSP70 were used for immunofluorescence studies in *I*. *scapularis* IDE8 or ISE6 cells. Uninfected and high percentage (80% infected cells) *A*. *phagocytophilum* (NY18)-infected or uninfected cells and low percentage (20% infected cells) and high percentage (80% infected cells) *A*. *phagocytophilum* (canine L610 isolate)-infected *I*. *scapularis* tick cells were used. The L610 isolate was provided by Erich Zweygarth, Lehrstuhl für Vergleichende Tropenmedizin und Parasitologie, Ludwig-Maximilians University, Munich, Germany. Cells were fixed with 10% neutral buffered formaldehyde. After 2 washes with PBS, 40 μl aliquots of cell suspension were placed on microscope slides and air-dried. The cells were permeabilized with 0.3% Triton-X100 in PBS for 30 min and 0.1% sodium dodecyl sulphate in PBS for 10 min, and washed 1x with PBS. After blocking with the CAS blocking agent (Invitrogen, Paisley, UK) for 1h, purified rabbit IgG (diluted 1: 100 to 1:300 in CAS blocking agent) was added and incubated overnight, followed by 3 washes in PBS. Incubation for 1 h with goat-anti-rabbit IgG conjugated with FITC (Abcam, Cambridge, UK) (diluted 1:1000 in CAS blocking agent) was followed by 3 washes in PBS. The slides were mounted in mounting medium containing DAPI (Vector Laboratories, Peterborough, UK). Images were acquired on an Axioskop 2 fluorescence microscope with an AxioCam MRc camera (Carl Zeiss, Cambridge, UK). Cytocentrifuge smears of *A*. *phagocytophilum*-infected and uninfected IDE8 cells were fixed for 3 min with technical methanol before staining for 20 min in Giemsa stain (10% v/v in deionised water buffered to pH 7.2). The percentage of cells infected with *A*. *phagocytophilum* was calculated by examining at least 200 cells using a 100x oil immersion objective. Images were acquired as described above.

### Immunofluorescence in adult female ticks

Adult female *I*. *scapularis* were infected with *A*. *phagocytophilum* (NY18) as described above. Female ticks were removed from the sheep 10 days after infestation, held in the humidity chamber for 4 days and fixed with 4% paraformaldehyde in 0.2M sodium cacodylate buffer, dehydrated in a graded series of ethanol and embedded in paraffin. Sections (4 μm) were prepared and mounted on glass slides. The paraffin was removed from the sections with xylene and the sections were hydrated by successive 2 min washes with a graded series of 100, 95, 80, 75 and 50% ethanol. The slides were treated with Proteinase K (Dako, Barcelona, Spain) for 7 min, washed with PBS and incubated with 3% bovine serum albumin (BSA; Sigma-Aldrich) in PBS for 1 h at room temperature. The slides were then incubated for 14 h at 4°C with primary antibodies diluted 1:100 to 1:300 in 3% BSA/PBS and after 3 washes in PBS developed for 1 h with goat-anti-rabbit IgG conjugated with FITC (Sigma-Aldrich) (diluted 1:160 in 3% BSA/PBS). The slides were washed twice with TBS and mounted in ProLong Antifade reagent (Molecular Probes, Eugene, OR, USA) or in mounting medium containing DAPI (Vector Laboratories, Peterborough, UK). The sections were examined using a Leica SP2 laser scanning confocal microscope (Leica, Wetzlar, Germany). Sections of uninfected ticks and IgGs from preimmune serum were used as controls.

### Immunofluorescence in recombinant *E*. *coli*

The *E*.*coli* induced for the production of recombinant *A*. *phagocytophilum* proteins as described before were fixed with 4% paraformaldehyde in PBS for 20 min at room temperature. *E*. *coli* cells producing recombinant tick Porin [15] were used as control. Bacterial smears were prepared using a cytocentrifuge. The cells were treated with 0.3% Triton X-100 in PBS for 30 min, blocked with 3% BSA in PBS for 1h at room temperature and incubated overnight at 4°C with purified antibodies (Pre-immune control, 35 μg ml^-1^; HSP70 and MSP4, 22 μg ml^-1^; GroEL, 30 μg ml^-1^) and developed as described previously for tick cells using goat-anti-rabbit IgG conjugated with FITC (Sigma; 1/160 dilution). The slides were washed twice with TBS and mounted in ProLong Antifade reagent (Molecular Probes). Images were acquired on a Nikon Eclipse Ti-U microscope with a 100x oil immersion objective and a Nikon Digital Sight DS Vi1 camera.

### Surface trypsin digestion of *A*. *phagocytophilum* and recombinant *E*. *coli*

The NY18 isolate of *A*.*phagocytophilum* was propagated in cultured ISE6 tick cells as described above. The *A phagocytophilum*-infected cells (approximately 1x10^7^ cells) were collected when 80%-90% of the cells were infected as determined by detection of intracellular morulae in stained cytospin cell smears. Host cell-free bacteria were isolated from cell lysates after five passages through a 27-gauge syringe, followed by differential centrifugation in Percoll gradients as previously described for *A*. *marginale* to separate bacteria from host cell debris [59]. The pellet of purified *A*. *phagocytophilum* was resuspended in 200 μl of SPG buffer (0.25 mM sucrose, 10 mM sodium phosphate, 5 mM L-glutamic acid, pH 7.2), and 5 μl of sequencing-grade trypsin (Promega) was added to half of the cell reaction mixture. Bacteria were incubated at 37°C for 30 min and then centrifuged at 10,000×g for 15 min and resuspended in Laemmli protein loading buffer, boiled for 5 min and loaded onto a 12% SDS-PAGE [21]. The *E*.*coli* induced for the production of recombinant *A*. *phagocytophilum* proteins (GroEL, MSP4, HSP70) were treated as described above for *A*. *phagocytophilum* and 10 μg protein were resuspended in Laemmli sample buffer and applied on 12% SDS-PAGE.

### Characterization of protein-protein interactions

Protein-protein interactions were characterized *in silico* using STRING 8.3 (http://string-db.org) and *in vitro* using recombinant MSP4, HSP70, GroEL proteins produced as described above. Tick mitochondrial Porin [15] was used as a control. Equimolar amounts of the recombinant HSP70, GroEL or Porin proteins were mixed in PBS and incubated overnight at 4°C. At the same time, 10 μl of Dynabeads Protein G slurry (Life Technologies/Thermo Fisher Scientific, Rocckford, IL, USA) were incubated with 10 μl of purified anti-HSP70 or anti-GroEL antibodies. The magnetic beads were then washed three times with PBS. The HSP70:GroEL and HSP70:Porin protein mixture was added and incubated for 2 h. Unbound proteins were removed and the beads were washed three times with PBS with addition of 0.1% Triton X-100 (Sigma). The beads were resuspended in Laemmli sample buffer, boiled for 5 min and loaded onto a 12% SDS-PAGE gel. After electrophoresis, proteins were transferred to a 0.2 μm nitrocellulose membrane (Bio-Rad). The membrane was blocked with 1% bovine serum albumin (BSA) for two hours at room temperature and incubated overnight at 4°C with primary antibodies (anti-HSP70, anti-GroEL or anti-Porin rabbit antibodies) at 1:200 dilution in 100 mM Tris HCL Buffer Saline (TBS). After incubation with the secondary anti-rabbit IgG peroxidase conjugated antibody (Sigma), protein bands were visualized with TMB stabilized substrate for horseradish peroxidase (Promega).

To characterize the interaction of recombinant HSP70 and GroEL with *A*. *phagocytophilum* proteins, a pellet of *A*. *phagocytophilum* purified as described above was used. Ten μl of Dynabeads Protein G slurry (Life Technologies/Thermo Fisher Scientific) were incubated with 10 μl of purified anti-HSP70, anti-GroEL or anti-Porin antibodies. The magnetic beads were then washed three times with PBS and 130 μg of *A*. *phagocytophilum* proteins were added and incubated for 2 hours. Unbound proteins were removed and the beads were washed three times with PBS with addition of 0.1% Triton X-100 (Sigma). The beads were resuspended in Laemmli sample buffer and loaded onto a 12% SDS-PAGE gel for Western blot analysis as described above.

For the characterization of MSP4 interactions with GroEL and HSP70, 3 aliquots of 100 μl of nickel beads slurry (Maxwell polyhistidine protein purification Kit, Promega, Madison, WI, USA) in 10 mM PBS (50:50) were incubated each with 120 μg of histidine-tagged MSP4 during 8 h at 4°C. After centrifugation, beads were washed with 1 ml of 10 mM PBS and incubated overnight with 30 μg of GroEL, HSP70 or Porin. After incubation and centrifugation, beads were washed with 1 ml of 10 mM PBS and proteins were eluted in Laemmli sample buffer, boiled for 5 min and loaded onto a 15% SDS-PAGE gel. After electrophoresis, proteins were stained with SyproRubi (BioRad, Hercules, CA, USA) and visualized after fluorescence scanning.

### Adhesion of recombinant *E*. *coli* strains to ISE6 tick cells

Adhesion of recombinant *E*. *coli* strains to ISE6 tick cells was tested as previously reported [27]. Briefly, *E*. *coli* strains were grown and induced as described before. *E*. *coli* strains with expression vector alone producing recombinant Thioredoxin and *A*. *marginale* MSP1a [27] were used as negative and positive control, respectively. Cell densities were determined and adjusted to 10^8^ cells ml^-1^ in LB. One hundred microlitres (10^7^ bacteria) culture were added to 900 ml of 10^6^ cells ml^-1^ suspensions of ISE6 tick cells in LB. Tick cells and bacteria were incubated for 30 min at 37°C with occasional agitation. Cells were then collected by centrifugation, washed two times in PBS and resuspended in 100 ml of PBS. Elimination of unbound bacteria from tick cells with bound bacteria was performed by Percoll (Sigma) gradient separation [27]. The band containing tick cells was removed with a pipette and washed in PBS. The final cell pellet was lysed in 1 ml of sterile water and 5 μl plated onto LB agar plates containing 100 μg of ampicillin per ml. Adhesive bacteria were quantitated as the number of colony forming units (CFU) recovered from each test and compared to the Thioredoxin control values by Student’s t-test for paired samples (P = 0.05).

### Electron microscopy

Cells were fixed in 2.5% glutaraldehyde in 0.1 M cacodylate buffer. Cells were rinsed in the cacodylate buffer, centrifuged and transferred into specimen carriers (1.2 mm in diameter, Leica). Then, 20% BSA was added and cells were immediately frozen using a Leica EM PACT2 high-pressure freezer. Freeze substitution (Leica EM ASF2) was carried in 2% osmium tetroxide diluted in 100% acetone at -90°C. After 96 h, material was warmed up to -20°C at a rate of 5°C/h and left for 24 h. Finally, the cells were warmed up again at the rate of 5°C/h to 4°C and left for 24 h. Pellets were removed from the carriers, rinsed three times in 100% acetone for 15 min each. Samples were infiltrated in graded series of SPI-pon resin (SPI) solutions (25%, 50%, 75%) diluted in acetone, 1 h at each step. After overnight incubation in pure resin, the samples were embedded in fresh resin solution and polymerized at 60°C for 48 h. Ultrathin sections were cut using an ultramicrotome Leica UCT, transferred to grids, double stained in ethanolic uranyl acetate for 30 min and lead citrate for 20 min. The samples were observed using a JEOL 1010 transmission electron microscope at an accelerating voltage of 80 kV. Images were captured with MegaView III camera (SIS GmbH).

For electron tomography, 10 nm gold nanoparticles coupled to Protein A (Aurion) were adsorbed to both sides of each section as fiducial markers and sections were carbon coated. Images were obtained using JEOL 2100F transmission electron microscope equipped with a high-tilt stage, Gatan camera (Orius SC 1000) and SerialEM automated acquisition software [60]. Tilt series images were collected as dual-axis tilt series over -60° to 60° tilt range along X-axis and -47° to 47° along Y-axis (increments 0.6 and 0.4, respectively) at pixel size of 0.554 nm. The IMOD software package was used for tomogram reconstruction.

### Antibody inhibition assay

The inhibitory effect of antibodies against the differentially represented surface-exposed proteins, GroEL, MSP4 and HSP70, on *A*. *phagocytophilum* (NY18) infection of ISE6 tick cells was conducted as described previously for *A*. *marginale* [37]. Confluent monolayers of ISE6 tick cells were pooled and used to seed 24-well plates for each assay. Each well received 1x10^6^ tick cells in L-15B medium 48 h prior to inoculation with *A*. *phagocytophilum*. Infected cultures for inoculum were harvested when monolayers were detaching (90% infected cells) and host cells were mechanically disrupted with a syringe and 26-gauge needle. Rabbit IgGs (2.2–2.4 mg/ml) were mixed with inoculum (1:1) for 60 min before being placed on the cell monolayers. Each monolayer then received 100 μl of the inoculum plus IgG mix and plates were incubated at 34°C for 30 min. The inoculum was removed from the wells and monolayers washed three times with PBS. Complete medium (1 ml) was added to each well and the plates were incubated at 34°C. Controls for each trial included (a) inoculum incubated with pre-immune IgG, (b) inoculum incubated with medium only (untreated infected control), (c) uninfected tick cells that served as background control, and (d) incubation with anti-tick Porin IgG. Four replicates were done for each treatment. After 7 days, monolayers from all wells were harvested, resuspended in 1 ml PBS and frozen at -70°C. Samples were thawed and solubilized with 1% Triton-X100 and processed for *A*. *phagocytophilum* detection by PCR after DNA extraction using TriReagent (Sigma) according to the manufacturer’s recommendations. *A*. *phagocytophilum* infection levels were determined by *Anaplasma 16S rDNA* and *msp4* real-time PCR normalizing against tick *16S* mitochondrial *rDNA* as described previously [7, 61] but using oligonucleotide primers MSP4-L (5’-CCTTGGCTGCAGCACCACCTG-3’) and MSP4-R (5’-TGCTGTGGGTCGTGACGCG3’) and PCR conditions of 5 min at 95°C and 35 cycles of 10 sec at 95°C, 30 sec at 55°C and 30 sec at 60°C. Results were compared between treatments by the Student’s t-test with unequal variance (P = 0.05).

### Analysis of mRNA levels by real-time RT-PCR

Real-time RT-PCR was performed on tick RNA samples obtained from low and high percentage *A*. *phagocytophilum* (NY18)-infected ISE6 tick cells (N = 5 for each time point) with gene-specific primers (Table 2) using the iScript One-Step RT-PCR Kit with SYBR Green and the iQ5 thermal cycler (Bio-Rad, Hercules, CA, USA) following manufacturer's recommendations. A dissociation curve was run at the end of the reaction to ensure that only one amplicon was formed and that the amplicons denatured consistently in the same temperature range for every sample [62]. The mRNA levels were normalized against tick *16S rRNA* [13] using the genNorm method (ddCT method as implemented by Bio-Rad iQ5 Standard Edition, Version 2.0) [63]. Normalized Ct values were compared between low and high percentage infected tick cells by Student's t-test with unequal variance (P = 0.05).

**Table 2:** Primer sequences and PCR conditions used for real-time RT-PCR.

Gene	Forward and reverse primers (5´-3´)	PCR annealing conditions
*groEL*	TGGTGGTGGGGCTGCATTGC ACGCATGGTGCTTCTTCAGAACC	62°C/30s
*msp4*	GCTGTGGGTCGTGACGCGAC CGCCCCTAACCCAGCACACA	62°C/30s
*hsp70*	CGTGATTACCGTGCCCGCGT GTCCGCTGCTTGTCGCCCTT	62°C/30s

### Prediction of T4SS effectors

The *A*. *phagocytophilum* proteins identified as over-represented in tick salivary glands and/or high percentage infected tick cells were subjected to S4TE software analysis to evaluate their potential as T4SS substrates. This software screens proteins for thirteen characteristic features of T4SS effectors [47]. S4TE also serves to visualize the distribution of predicted T4SS effectors relative to whole genome architecture [47].

## Supporting Information

S1 Fig Western blot analysis of the specificity of antibodies produced in rabbits against *A*. *phagocytophilum* recombinant proteins. Western blot analysis of 10 μg of recombinant *A*. *phagocytophilum* MSP4, GroEL and HSP70 proteins produced in *E*. *coli* (red arrows) demonstrates the specificity of the antibodies produced in rabbits. *E*. *coli* cell proteins were included in two lanes (x2) as negative control. Abbreviation: MW, molecular weight markers (Spectra multicolor broad range protein ladder; Thermo Scientific).
(PDF)

S2 Fig Characterization of the possible role of *A*. *phagocytophilum* type IV secretion system during pathogen infection. (A) Characterization of the T4SS proteins in infected tick guts and salivary glands. Only peptides with a confidence of at least 99% and identified with two or more PSM in at least one of the samples were considered to perform protein quantification. Proteins with the same description in the Anaplasmataceae were grouped and the total number of PSM for each protein were normalized against the total number of PSM on each infected adult female tick tissue and compared between salivary glands and guts by Chi2-test (*P<0.05). Results are the mean of two replicates. (B) Analysis of T4SS effector features for the *A*. *phagocytophilum* Iron-binding protein (APH_0051), HSP70 (APH_0346) and Peptidase (APH_1159) using S4TE software. Only positive hits are shown and corresponding domains or features were highlighted. The sequences of identified Nuclear Localization Signals, E-block and Coiled-coil domains are also indicated.
(PDF)

S1 Table *A*. *phagocytophilum* proteins in low and high percentage infected ISE6 tick cells. (XLSX)

S2 Table *A*. *phagocytophilum* proteins in infected *I*. *scapularis* female tick guts and salivary glands. (XLSX)

S1 Video Electron tomography with a colored model of *E*. *coli* producing *A*. *phagocytophilum* MSP4 that are phagocytized by ISE6 tick cells. Membranes of both cells are in tight contact in several places. Color code: outer membrane of recombinant *E*. *coli* (green), plasma membrane of ISE6 tick cells (blue), small vesicles probably originating from the outer membrane (red and arrow).
(MOV)
