# Supplementary material for: Identification and Characterization of Anaplasma phagocytophilum Proteins Involved in Infection of the Tick Vector, Ixodes scapularis
Source: PLoS One. 2015 Sep 4;10(9):e0137237. doi: 10.1371/journal.pone.0137237 (PMC4560377; doi:10.1371/journal.pone.0137237)
Supplement: S1 Fig — Western blot analysis of 10 μg of recombinant A. phagocytophilum MSP4, GroEL and HSP70 proteins produced in E. coli (red arrows) demonstrates the specificity of the antibodies produced in rabbits. E. coli cell proteins were included in two lanes (x2) as negative control. Abbreviation: MW, molecular weight markers (Spectra multicolor broad range protein ladder; Thermo Scientific). (PDF) [file pone.0137237.s001.pdf]

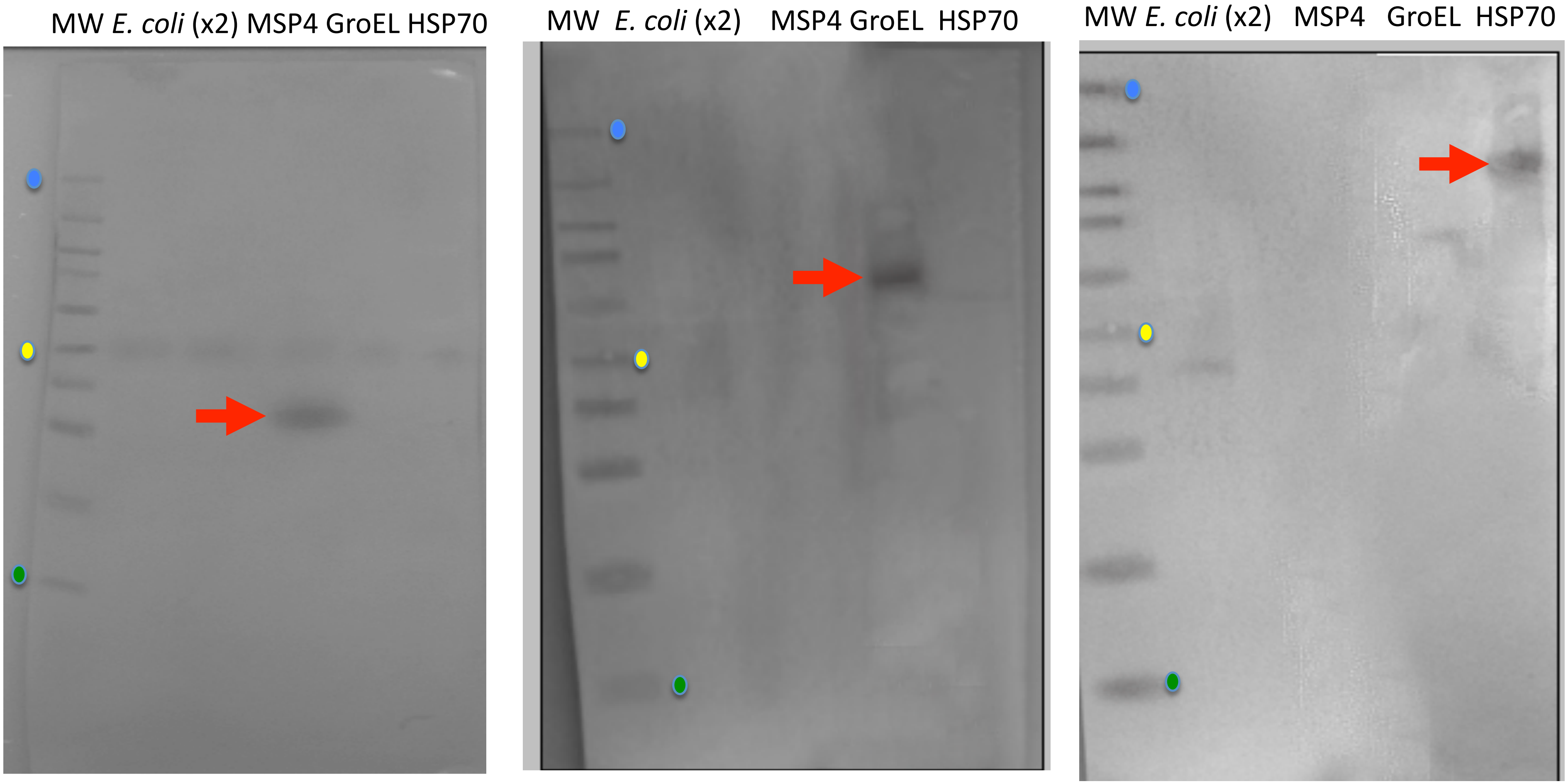

Anti-MSP4 antibodies

Anti-GroEL antibodies

Anti-HSP70 antibodies

260 kDa

40 kDa

10 kDa

MW: Molecular weight markers (Spectra multicolor broad range protein ladder; Thermo Scientific.
