# Supplementary figures and images for: Identification and Characterization of Anaplasma phagocytophilum Proteins Involved in Infection of the Tick Vector, Ixodes scapularis
Source: PLoS One. 2015 Sep 4;10(9):e0137237. doi: 10.1371/journal.pone.0137237 (PMC4560377; doi:10.1371/journal.pone.0137237)

**A**

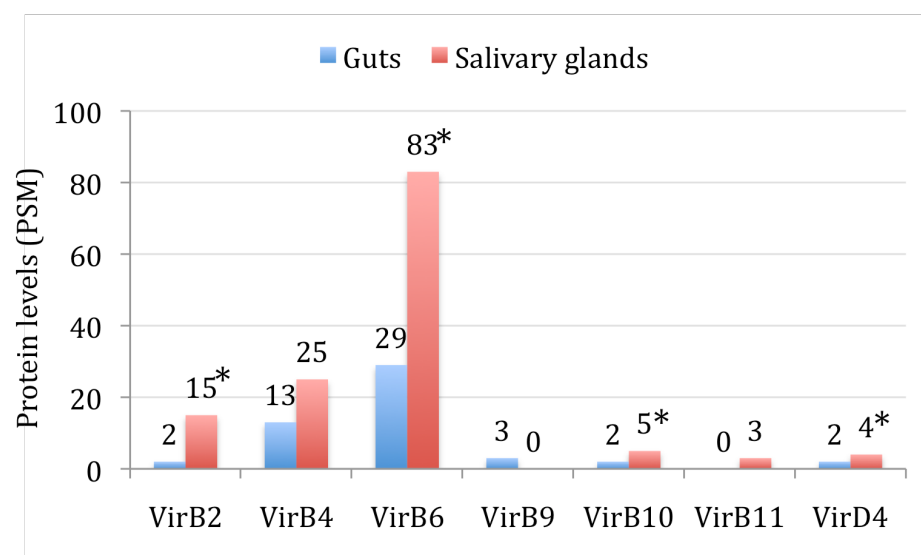

B

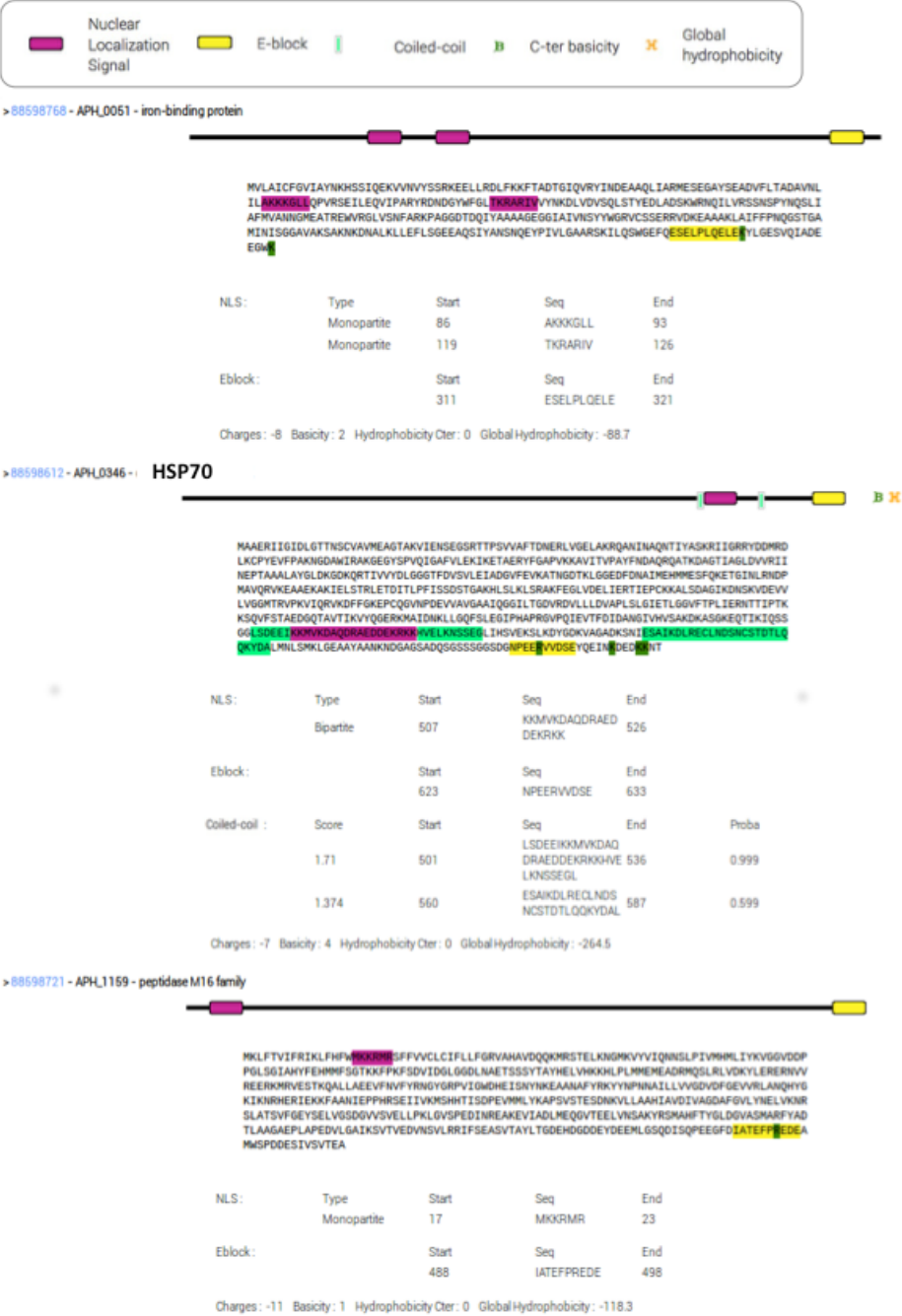

Supplement: S2 Fig — (A) Characterization of the T4SS proteins in infected tick guts and salivary glands. Only peptides with a confidence of at least 99% and identified with two or more PSM in at least one of the samples were considered to perform protein quantification. Proteins with the same description in the Anaplasmataceae were grouped and the total number of PSM for each protein were normalized against the total number of PSM on each infected adult female tick tissue and compared between salivary glands and guts by Chi2-test (*P<0.05). Results are the mean of two replicates. (B) Analysis of T4SS effector features for the A. phagocytophilum Iron-binding protein (APH_0051), HSP70 (APH_0346) and Peptidase (APH_1159) using S4TE software. Only positive hits are shown and corresponding domains or features were highlighted. The sequences of identified Nuclear Localization Signals, E-block and Coiled-coil domains are also indicated. (PDF) [file pone.0137237.s002.pdf]
